# Identification of mRNA vaccines and conserved ferroptosis related immune landscape for individual precision treatment in bladder cancer

**DOI:** 10.1186/s40537-022-00641-z

**Published:** 2022-07-07

**Authors:** Cheng-Peng Gui, Jia-Ying Li, Liang-Min Fu, Cheng-Gong Luo, Chi Zhang, Yi-Ming Tang, Li-zhen Zhang, Guan-nan Shu, Rong-Pei Wu, Jun-Hang Luo

**Affiliations:** 1 Department of Urology, First Affiliated Hospital, Sun Yat-Sen University, Guangzhou, 510080 Guangdong China; 2 Institute of Precision Medicine, First Affiliated Hospital, Sun Yat-Sen University, Guangzhou, Guangdong China; 3 Department of Urology, Third Affiliated Hospital, Sun Yat-Sen University, Guangzhou, Guangdong China; 4 Department of Urology, Second Affiliated Hospital, Guangzhou Medical University, Guangzhou, Guangdong China

**Keywords:** Ferroptosis, Bladder cancer, Precise treatment, mRNA vaccine, Immunotherapy, Tumor immune microenvironment

## Abstract

**Background:**

The aim of this study was to identify the ferroptosis induced tumor microenvironment (FeME) landscape in bladder cancer (BCa) for mRNA vaccine development and selecting suitable patients for precision treatment.

**Methods:**

Gene expression profiles and clinical information of 1216 BCa patients were extracted from TCGA-BLCA, three GEO databases and IMvigor210 cohort. We comprehensively established the FeME landscape of 1216 BCa samples based on 290 ferroptosis related genes (FRGs), and systematically correlated these regulation patterns with TME cell-infiltrating characteristics. Besides, we identified the patients’ ferroptosis risk index (FRI) to predict the prognosis of BCa for precise treatment.

**Results:**

Six over-expressed and mutated tumor antigens associated with poor prognosis and infiltration of antigen presenting cells were identified in BCa. Furthermore, we demonstrated the evaluation of FeME within individual tumors could predict stages of tumor inflammation, subtypes, genetic variation, and patient prognosis. Then, 5-lncRNA signature was mined to produce the FRI. Low FRI was also linked to increased mutation load, better prognosis and enhanced response to anti-PD-L1 immunotherapy. Besides, an immunotherapy cohort confirmed patients with lower FRI demonstrated significant therapeutic advantages and clinical benefits.

**Conclusions:**

TFRC, SCD, G6PD, FADS2, SQLE, and SLC3A2 are potent antigens for developing anti-BCa mRNA vaccine. Establishment of FRI will contribute to enhancing our cognition of TME infiltration characterization and guiding more effective immunotherapy strategies and selecting appropriate patients for tumor vaccine therapy.

**Supplementary Information:**

The online version contains supplementary material available at 10.1186/s40537-022-00641-z.

## Background

BCa is one of the most common malignancies worldwide, with high morbidity and mortality [1]. More than 500,000 new BCa cases and 200,000 BCa-related deaths occur worldwide annually [2]. BCa has two main subtypes: muscle-invasive BCa and nonmuscle-invasive BCa. Although the 5-year survival rate of nonmuscle-invasive BCa is about 90%, approximately 15–20% of such cases would progress to the muscle-invasive stage and even to distant metastasis, which has a dismal 5-year survival rate of 5–30% [1].

In the last decade, some large-scale clinical trials, such as KEYNOTE-045 and IMvigor211, demonstrated that BCa is susceptible to ICIs, representing an important advancement in the treatment of BCa [3, 4]. When patients can’t afford immunotherapy, there may be another treatment that works. Recently, tumor vaccines targeting the ganglioside GD2 and CA-199/KLH have been successful in mitigating pancreatic adenocarcinoma progression [5, 6]. Although these vaccines provided a survival benefit of only several months, the results are encouraging enough to explore the potential of anti-tumor vaccines. Prototypical cancer vaccines have the advantages of relative non-toxicity, minimal nonspecific effects, broad therapeutic window and induction of persistent immunological memory [7, 8]. Therefore, cancer vaccines can overcome the drug resistance, adverse reactions, limited therapeutic efficacy and high costs associated with standard chemo- and immunotherapies [7]. However, no mRNA vaccine against bladder cancer antigens has been developed so far, and no subgroup of patients suitable for vaccination has been identified.

Simultaneously, ferroptosis, a form of iron-dependent and nonapoptotic cell death, is attracting increasing attention in view of the fact that apoptosis resistance is one of the hallmarks of tumors [9]. Inducing tumor cell ferroptosis seems to be an attractive and promising therapeutic strategy, especially for drug resistant malignancies. Recent evidence has revealed a strong association between ferroptosis and tumor immune reactions [10, 11]. For instance, Wang et al. found that IFNγ released from CD8+ T cells could suppress the expression of SLC7A11, thereby promoting lipid peroxidation in cancer cells and inducing ferroptosis [10]. The above suggested that the combination of mRNA vaccines and ferroptosis inducers is a promising treatment.

Long noncoding RNAs (lncRNAs), a type of RNA molecule with transcripts of > 200 nucleotides, participate in tumorigenesis and cancer development not only by altering the malignancy of cancer cells themselves but also by changing the TME, as reported in many studies [12]. In recent years, the interaction between lncRNAs and ferroptosis has also been investigated. For instance, the lncRNA P53RRA serves as a tumor suppressor by promoting p53 maintenance in the nucleus, thus, facilitating ferroptosis [13].

In this study, we aimed to verify the close association between ferroptosis and the TME and to propose an important tool for predicting the prognosis and immune infiltration of BCa, also for precise treatment of immunotherapy and developing mRNA vaccines.

## Methods

### Bladder cancer dataset source and preprocessing

**Fig. 1:**
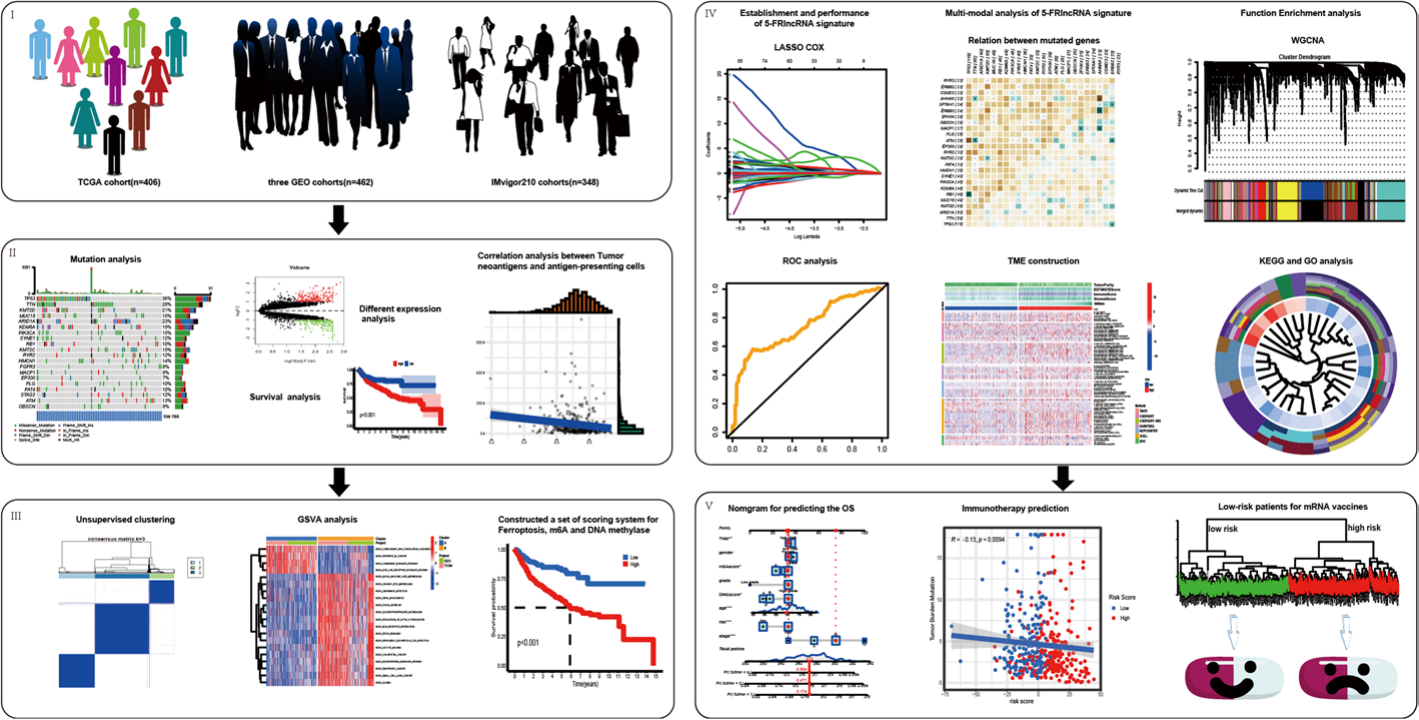
The flow chart of the study procedure

The workflow of our study was shown in Fig. 1. Public RNA-seq data and full clinical annotation were downloaded from Gene-Expression Omnibus (GEO) and the Cancer Genome Atlas (TCGA) database. Patients without survival information were removed from further evaluation. In total, three eligible BCa cohorts (GSE13507, GSE32894 and GSE48075) and TCGA-BLCA (The Cancer Genome Atlas-Bladder Cancer) cohort were gathered in this study for further analysis. For microarray data from GEO database, the normalized matrix files were directly downloaded. As to datasets in TCGA, RNA sequencing data (FPKM value) of gene expression were downloaded from the Genomic Data Commons (GDC, https://portal.gdc.cancer.gov/). And then FPKM values were transformed into transcripts per kilobase million (TPM) values. Batch effects from non-biological technical biases were corrected using the “ComBat” algorithm of sva package. The baseline information of all eligible BCa datasets was summarized in Table 1. The somatic mutation data was acquired from TCGA database.

**Table 1:** Clinical features of all eligible 1216 BCa patients from TCGA, GEO and IMvigor210 cohorts

Variables	TCGA cohort	GEO cohort	IMvigor210 cohort	
	(n = 406)	(n = 462)	(n = 348)	
Status				
Alive	298 (73.40)	340 (73.59)	116 (33.33)	
Dead	108 (26.60)	122 (26.41)	232 (66.67)	
Age	68.09 ± 10.57	67.81 ± 11.51	NA	
Gender				
Female	107 (26.35)	111 (24.03)	76 (21.84)	
Male	299 (73.65)	351 (75.97)	272 (78.16)	
AJCC—T			Response:CR	25(7.18)
T1	11(2.70)	277(59.96)		
T2	193(47.54)	116(25.11)	PR	43(12.36)
T3	158(38.92)	49(10.61)	SD	63(18.10)
T4	44(10.84)	20(4.32)	PD	167(47.99)
			NE	50(14.37)
AJCC—N			Immune phenotype:inflammed	74(21.26)
N0	239(58.86)	414(89.61)		
N1	47(11.58)	48(10.39)	excluded	134(38.51)
N2	76(18.72)		desert	76(21.84)
N3	8(1.97)			
Nx	36(8.87)		NE	64(18.39)
AJCC—M				
M0	196(48.28)	448(96.97)		
Mx/M1	210(51.72)	14(3.03)		
Stage I	2(0.49)	NA	118(33.90)	
II	128(31.53)	NA	95 (27.30)	
III	140(34.48)	NA	69 (19.833)	
IV	136(33.50)	NA	66 (18.97)	
Tumor_grade				
High grade	382(94.09)	155(33.55)	NA	
Low grade	21(5.17)	234(50.65)	NA	
Unknown	3(0.74)	73(15.80)	NA	
Median follow-up,months(IQR)	17.73(10.83–31.60)	35.54(16.72–62.13)	8.05(2.87–17.87)	
Overall survival (95% CI)				
1 years	85.2(83.4–87.0)	92.0(90.7–93.3)	3 months	76.0(73.7–78.3)
3 years	66.2(63.1–69.3)	80.2(78.2–82.2)	6 months	60.7(58.7–63.4)
5 years	61.3(57.8–64.8)	70.3(67.6–73.0)	9 months	49.3(46.6–52.0)
			12 months	41.2(38.5–43.9)

We identified a 5-lncRNA signature in TCGA cohort, and verified this signature in GEO cohort. Besides, we demonstrated significant therapeutic advantages and clinical benefits in an immunotherapy cohort (IMvigor210 cohort)

Data are shown as n(%)

IMvigor210 cohort downloaded from http://research-pub.gene.com/IMvigor210CoreBiologies/packageVersions/

AJCC, American Joint Committee on Cancer; GEO, Gene Expression Omnibus; BCa, bladder cancer; TCGA, The Cancer Genome Atlas

Besides, we performed a systematical search for the immune checkpoint blockade gene expression profiles, which could be publicly obtained and reported with complete clinical information. An immunotherapeutic cohort was finally included in our study: advanced urothelial cancer with intervention of atezolizumab, an anti-PD-L1 antibody (IMvigor210 cohort) [14]. For IMvigor210 cohort, based on the Creative Commons 3.0 License, the complete expression data and detailed clinical annotations could be obtained from http://research-pub.Gene.com/imvigor210corebiologies. The raw count data were normalized by the DEseq2 R package and then the count value was transformed into the TPM value.

Additionally, a total of 290 ferroptosis regulators were identified from a previous study [9]. To select lncRNAs, the annotation file was obtained from the Ensembl database (http://asia.ensembl.org).

### Different expression analysis and Kaplan–Meier survival analysis

Differential gene expression and patient survival data were integrated using Gene Expression Profiling Interactive Analysis [15] (GEPIA, http://gepia2.cancer-pku.cn, version 2). ANOVA was used to identify the differentially expressed genes with |log_2_FC| values > 1 and q values < 0.01. OS and disease-free survival (DFS) were evaluated using the Kaplan–Meier method with a 50% (Median) cutoff, and compared by the log rank test. The cox proportional hazards regression model was applied to calculate the hazard ratio. The parameter setting was consistent in each analysis without adjustment for any p value. One-way ANOVA and Kruskal–Wallis tests were used to conduct difference comparisons of three or more groups [16]. p-values < 0.05 were considered statistically significant.

### Comparison of genetic alterations and estimation of tumor immune cell infiltration

The cBio Cancer Genomics Portal [17] (cBioPortal, http://www.cbioportal.org) was used to integrate the raw RNA-seq data from TCGA and other databases, and compare genetic alterations in Bladder Cancer. p-values < 0.05 were considered statistically significant. Tumor Immune Estimation Resource [18] (TIMER, https://cistrome.shinyapps.io/timer/) was used to analyze and visualize the association between abundance of tumor immune infiltrating cells (TIICs) and ferroptosis related genes through analytical modules for gene expression, somatic mutations, clinical outcomes and somatic copy number alteration. Purity Adjustment was selected using Spearman’s correlation analysis. p-values < 0.05 were considered statistically significant. We also used the ssGSEA (single-sample gene-set enrichment analysis) algorithm to quantify the relative abundance of each cell infiltration in the BCa TME. The enrichment scores calculated by ssGSEA analysis were utilized to represent the relative abundance of each TME infiltrating cell in each sample.

### Unsupervised clustering for 55 FRGs

A total of 55 regulators were extracted from three integrated GEO datasets and TCGA cohorts for identifying distinct ferroptosis regulation patterns mediated by FRGs. Unsupervised clustering analysis was applied to identify distinct ferroptosis regulation patterns based on the expression of 55 FRGs and classify patients for further analysis. The number of clusters and their stability were determined by the consensus clustering algorithm [19]. We used the “ConsensusClusterPlus” package to perform the above steps and 1000 times repetitions were conducted for guaranteeing the stability of classification [20]. And we verified the unsupervised clustering analysis in another external database (IMvigor210 cohort).

### Gene set variation analysis (GSVA) and functional annotation

To investigate the difference on biological process between ferroptosis regulation patterns, we performed GSVA enrichment analysis using “GSVA” R packages. GSVA, in a non-parametric and unsupervised method, is commonly employed for estimating the variation in pathway and biological process activity in the samples of an expression dataset [21]. The gene sets of “c2.cp.kegg.v6.2.-symbols” were downloaded from MSigDB database for running GSVA analysis. Adjusted p-value less than 0.05 was considered as statistically significance. The clusterProfiler R package was used to perform functional annotation for ferroptosis-related genes, with the cutoff value of FDR < 0.05.

### Generation of comprehensive ferroptosis related genes score

As previously mentioned, we classified patients into three distinct ferroptosis regulation patterns based on the expression of 55 FRGs. The empirical Bayesian approach of limma R package was applied to determine DEGs between different regulation patterns [22]. The significance criteria for determining DEGs was set as adjusted p-value < 0.001. And to understand the potential functions of these developed ferroptosis regulation patterns, gene ontology (GO) term enrichment analysis and Kyoto Encyclopedia of Genes and Genomes (KEGG) pathway analysis were performed in the Enrichr database (http://amp.pharm.mssm.edu/Enrichr/).

Then we further quantified the ferroptosis regulation patterns of individual tumor, and constructed a set of scoring system to evaluate the ferroptosis pattern of individual patients with BCa—the ferroptosis related genes score, and we termed as FRGs score. The procedures for establishment of FRGs score were as follows:

The DEGs identified from different ferroptosis regulation patterns were firstly normalized among all samples and the overlap genes were extracted. The patients were classified into several groups for deeper analysis by adopting unsupervised clustering method for analyzing overlap DEGs. The consensus clustering algorithm was utilized for defining the number of gene clusters as well as their stability. Then, we performed the prognostic analysis for each gene in the signature using univariate Cox regression model. The genes with the significant prognosis were extracted for further analysis. We then conducted principal component analysis (PCA) to calculate FRGs score. This method had advantage of focusing the score on the set with the largest block of well correlated (or anticorrelated) genes in the set, while down-weighting contributions from genes that do not track with other set members. We then define the FRGs score using a method similar to GGI [23, 24]:

$$ {\text{FRGs score}} = \sum {\left( {{\text{PC1}}_{{\text{i}}} + {\text{PC2}}_{{\text{i}}} } \right)} , $$

where i is the expression of ferroptosis patterns-related genes.

### Further establishment of a convenient and brief ferroptosis related lncRNA signature

First, 607 Fr-lncRNAs(ferroptosis related lncRNAs) were obtained through different expression analysis between groups of high FRGs score and low FRGs score by limma package(lncRNAs with an average expression value of < 0.05 and |logFC| > 2). Then, after comparison of expression of genes between 19 normal-tissue samples and 406 BCa samples, 346 lncRNAs were obtained by the Wilcoxon test (|logFC| > 1). Next, univariate Cox survival analysis was employed to explore relationships between OS and 346 differentially expressed lncRNAs linked to ferroptosis in the training set of TCGA cohort. The HR and p-value were generated using the “survival package” in R. Once significance (p < 0.001) had been reached, 44 lncRNAs were selected for use in the LASSO method to search for the hub lncRNAs related to survival. And we drew a plot with the partial likelihood deviance versus log (λ) (with λ representing a “tuning” parameter) (Fig. 6A). Based on this process, six important prognostic lncRNAs were selected from prognostic lncRNAs.

Next, multivariate Cox regression analysis (MCRA) was used to estimate the regression coefficient of less important prognostic lncRNAs associated with ferroptosis in the training set. A 5-lncRNA signature comprising these selected lncRNAs associated with ferroptosis was constructed using respective coefficients. According to the ferroptosis risk index (FRI) formula, FRI of each patient was calculated, and the median FRI was determined using “survminer” in R to classify patients in the training set into a low-risk group and high-risk group. Then, differences in OS between the low-risk group and high-risk group were compared using the Kaplan–Meier method. To test the sensitivity and specificity of the FRI formula, ROC curves were created to assess the predictive accuracy. Similarly, the formula was applied in the test set to validate its stability. Clinicopathologic characteristics (e.g., TNM stage) are closely associated with the prognosis of BCa patients. Hence, univariate and MCRA were undertaken to test whether the FRI was independent of clinicopathologic characteristics.

ANOVA was used to determine the association of immune subtypes with different immune-related molecular and cellular characteristics. The most frequently mutated genes were screened using the chi-square test. DAVID program was used to functionally annotate each gene module through gene ontology analysis. Single-sample GSEA (ssGSEA) was used to calculate immune enrichment scores for each sample, which is the measure of genes that are coordinately up- or down-regulated within a sample. We also conducted validation analysis in the IMvigor210 cohort using these identified crucial lncRNAs.

### WGCNA analyses

To explore relationships between the lncRNAs signature and biological functions of BCa, WGCNA was employed to construct gene co-expression modules among differentially expressed mRNAs [25]. Modules with the maximum significance associated with the lncRNAs signature were selected. Then, enrichment analyses of these genes in the most highly related module were done using the “clusterProfiler” package in R software.

### Predicting the effective response of postoperative immunotherapy and evaluated the immunotherapy response in IMvigor210 cohort

We downloaded the IPS of ccRCC patients from the cancer immune group atlas (TCIA) (https://tcia.at/home). The patient’s IPS was obtained without bias by considering the four categories of immunogenicity-determining genes: effector cells, immunosuppressor cells, MHC molecules and immune modulators. This step was performed by evaluating gene expression in four cell types and the IPS was calculated on a scale of 0–10 based on z-scores representing gene expression in cell types. Higher IPS scores are positively correlated to the increased immunogenicity [26]. Meanwhile, the TIDE algorithm was used to predict ICB responses and evaluate ability to serve as a neoantigen (http://tide.dfci.harvard.edu) [27]. Then, we calculate the FRI in cohort of advanced urothelial cancer with intervention of atezolizumab, an anti-PD-L1 antibody (IMvigor210 cohort), and explore the effect of FRI on immunotherapy response.

### Nomogram construction based on the FRI and comparison of the predictive ability with clinicopathologic characteristics

A nomogram comprising independent prognostic factors was developed to predict 1-, 3-, and 5-year OS rates by employing the “rms” package in R. The capacity of the nomogram to distinguish survival was tested using AUC values. The DCA curve indicated that a nomogram was feasible to make valuable and profitable judgments. Furthermore, among these detected factors included in the nomogram, we also observed that “FRI” was more beneficial than the other clinical and laboratory indicators in the prediction of overall survival.

### Statistical analysis

All data sorting and analyses were completed by the R 4.1.0 software. For continuous variables with normal distribution and homogeneity of variance, an independent sample t-test was used; otherwise, Wilcoxon rank-sum test was selected. Pearson correlation coefficient test was used to analyze the correlation. A value of p < 0.05 was considered significant.

## Results

### Identification of potential ferroptosis related mRNA vaccines of BCa

**Fig. 2:**
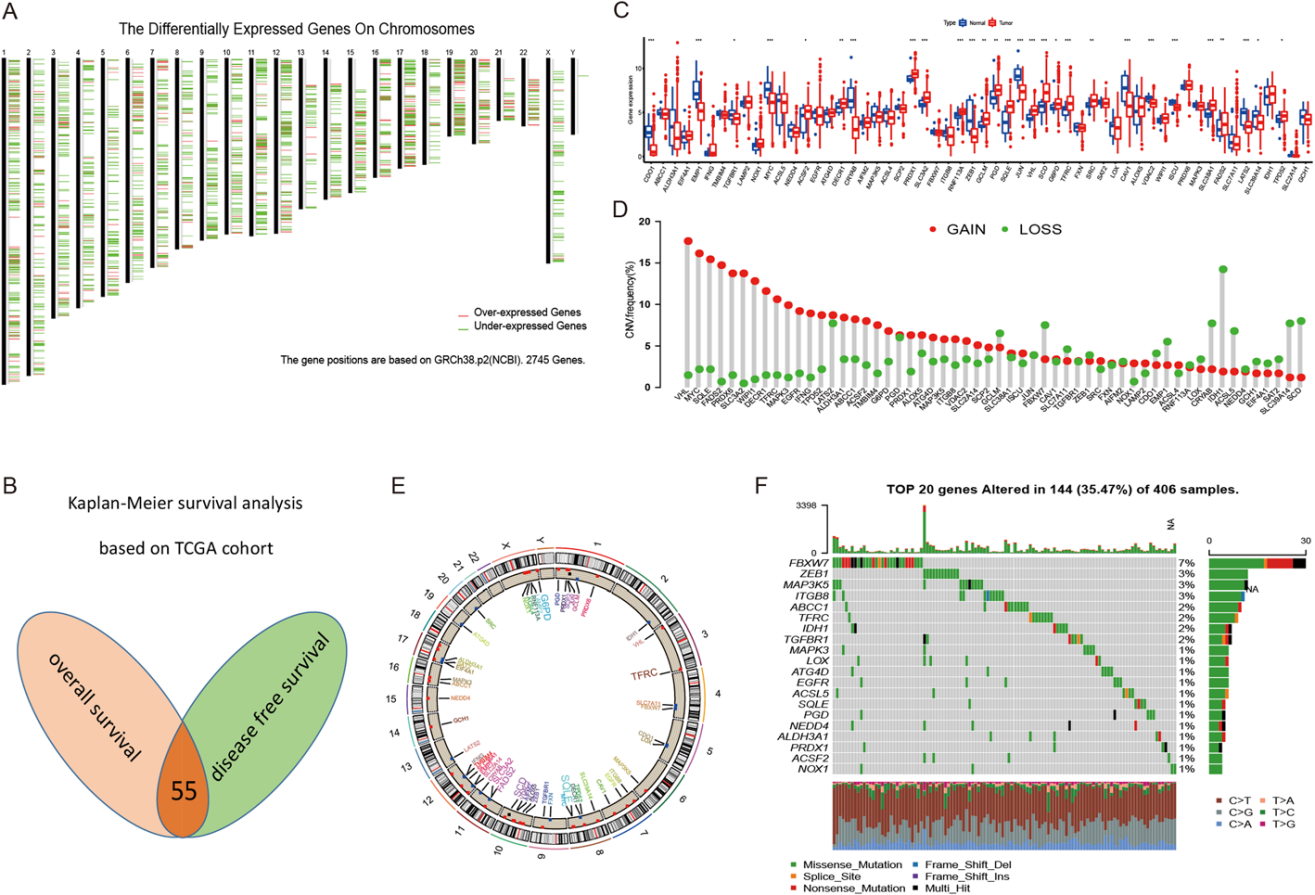
Identification of potential tumor antigens and landscape of genetic and expression variation of ferroptosis regulators in bladder cancer. **A** Potential tumor-associated antigens of BCa. **B** Overlap genes of univariate Cox regression analysis based on overall survival and disease-free survival. **C** The expression of ferroptosis regulators between normal tissues and BCa tissues. (*p < 0.05; **p < 0.01; ***p < 0.001). **D** The CNV variation frequency of ferroptosis regulators in TCGA-BLCA cohort. **E** The location of CNV alteration. **F** The mutation frequency of top20 ferroptosis regulators

**Fig. 3:**
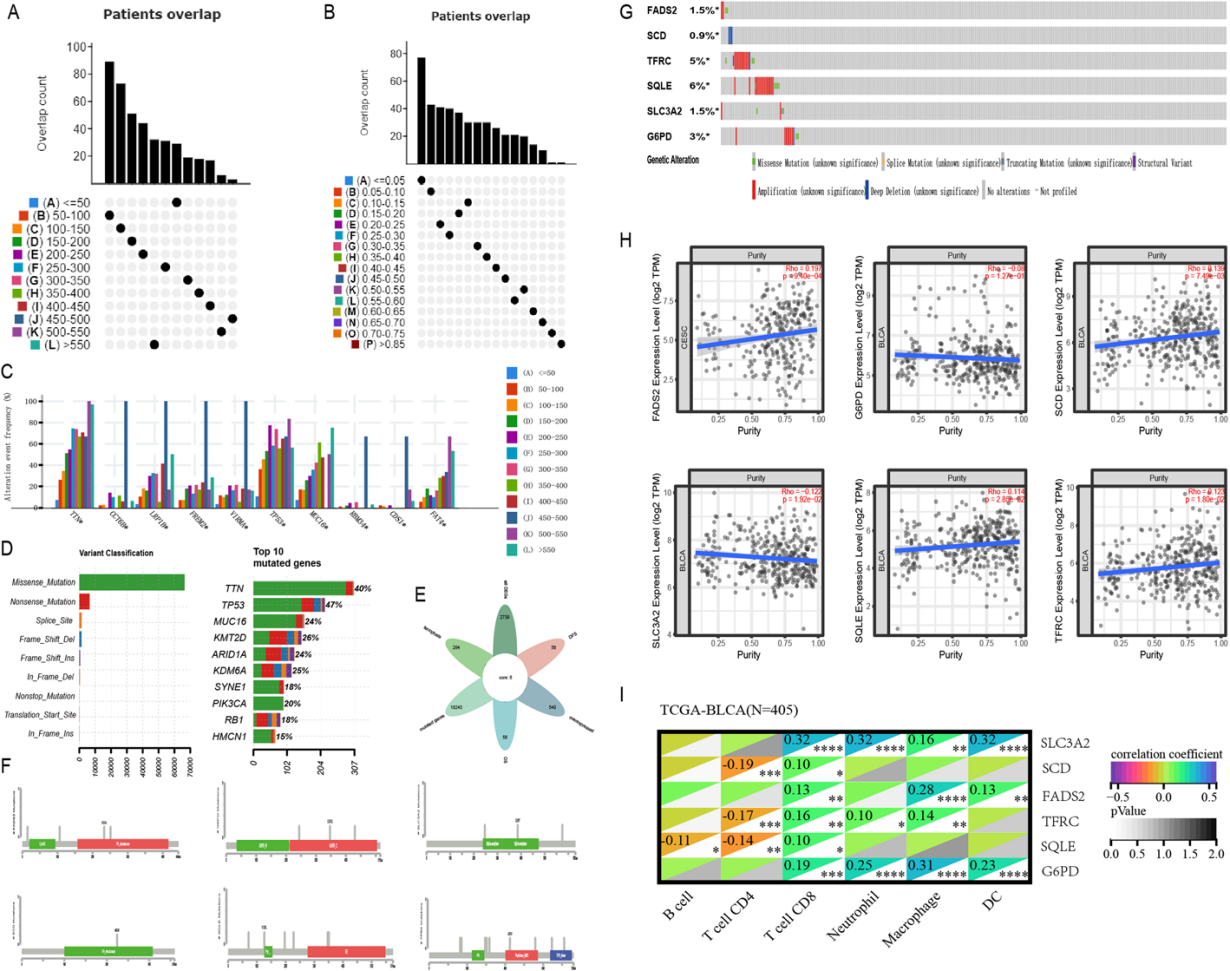
Identification of potential tumor-specific antigens of BCa. Samples overlapping in **A** mutation count groups and **B** altered genome fraction. **C** Genes with highest frequency in mutation count groups. **D** Summary of overall mutation results. **E** 6 candidates with high expression, mutation and significant association with OS and DFS. **F**, **G** The OncoPrint tab summarizes genomic alterations across TCGA set. **H**, **I** Identification of tumor antigens associated with tumor purity (**H**) and APCs (**I**)

To identify potential mRNA vaccines of BCa, we first screened for the aberrantly expressed genes and detected 546 overexpressed genes that likely encode tumor-associated antigens (Fig. 2A, 3E). A total of 18,249 mutated genes potentially encoding for tumor-specific antigens were then filtered by analyzing altered genome fraction and mutation counts in individual samples (Fig. 3A–E). Mutational analysis showed that titin (TTN) and tumor protein p53 (p53) were the most frequently mutated genes in terms of both altered genome fraction and mutation counts (Fig. 3C, D). Of note, in addition to TTN, p53 and mucin 16 (MUC16) in top 3 candidates with altered genome fractions, KMT2D, ARID1A, KDM6A, SYNE1, PIK3CA, RB1, as well as HMCN1, all have a mutation frequency of more than 15%, indicating the potential genomic interaction among them (Fig. 3D). According to the analysis, missense mutations had the highest incidence of all mutation types. High missense mutation counts were observed in TTN, p53, MUC16, KMT2D, ARID1A, KDM6A, SYNE1, PIK3CA, RB1 and HMCN1 (Fig. 3D). Overall, 534 overexpressed and frequently mutated tumor specific genes were identified.

After survival analysis about overall survival and DFS, a total of 55 FRGs from 290 FRGs were finally identified in this study (Fig. 2B). We first summarized the incidence of copy number variations and somatic mutations of 55 FRGs in BCa. Among 406 samples, 144 experienced mutations of FRGs, with frequency 34.95%. It was found that the FBXW7 exhibited the highest mutation frequency followed by ZEB1, SCD, FADS2, G6PD as well as SLC3A2 (Fig. 2F). Further analyses revealed a significant mutation cooccurrence relationship between FBXW7 and MAPK3, ZEB1 and ACSF2, along with PGD and EGFR (Additional file 1: Fig. S2B). The investigation of CNV alteration frequency showed a prevalent CNV alteration in 55 FRGs and most were focused on the amplification in copy number, while IDH1, FBXW7, CRYAB and SCD had a widespread frequency of CNV deletion (Fig. 2D). The location of CNV alteration of FRGs on chromosomes was shown in Fig. 2E. Based on the expression of these 55 FRGs, we could completely distinguished BCa samples from normal samples (Fig. 2C). To ascertain whether the above genetic variations influenced the expression of FRGs in BCa patients, we investigated the mRNA expression levels of regulators between normal and BCa samples, and found that the alterations of CNV could be the prominent factors resulting in perturbations on the FRGs expression. Compared to normal bladder tissues, FRGs with amplificated CNV demonstrated markedly higher expression in BCa tissues (e.g., VHL and SQLE), and vice versa (e.g., SLC39A14, CDO1, CRYAB, JUN and EMP1) (Fig. 2C, D). The above analyses presented the highly heterogeneity of genetic and expressional alteration landscape in FRGs between normal and BCa samples, indicating that the expression imbalance of FRGs played a crucial role in the BCa occurrence and progression.

The prognosis-related tumor antigens were next screened from the aforementioned genes as potential candidates for developing mRNA vaccine. Fifty-five genes were closely associated with the OS of BCa patients, of which 6 genes showed significant correlation with the DFS (Fig. 3E, Additional file 1: Fig. S1A). As shown in Additional file 1: Fig. S1A, patients overexpressing fatty acid desaturase 2(FADS2) in the tumor tissues had significantly shorter survival compared to the FADS2 low group. Likewise, high expression levels of stearoyl-CoA desaturase (SCD), squalene epoxidase (SQLE), glucose-6-phosphate dehydrogenase (G6PD), transferrin receptor (TFRC), solute carrier family 3 member 2 (SLC3A2) were also associated with poor prognosis (Additional file 1: Fig. S1A). Taken together, 6 gene candidates were identified that are critical for BCa development and progression. Furthermore, lower expression levels of SCD, SQLE, FADS2 and TFRC were significantly associated with increased tumor infiltration of DCs and/or B cells (Fig. 3H, I). G6PD and SLC3A2 exhibited the upregulated tendency in increased infiltration of Macrophages (Fig. 3H, I). These findings suggest that the identified tumor antigens may be directly processed and presented by the antigen presenting cells (APCs) to T cells, and recognized by the B cells to trigger an immune response, and are therefore promising candidates for developing mRNA vaccine against BCa.

### Identification of three ferroptosis regulation patterns mediated by 55 FRGs

**Fig. 4:**
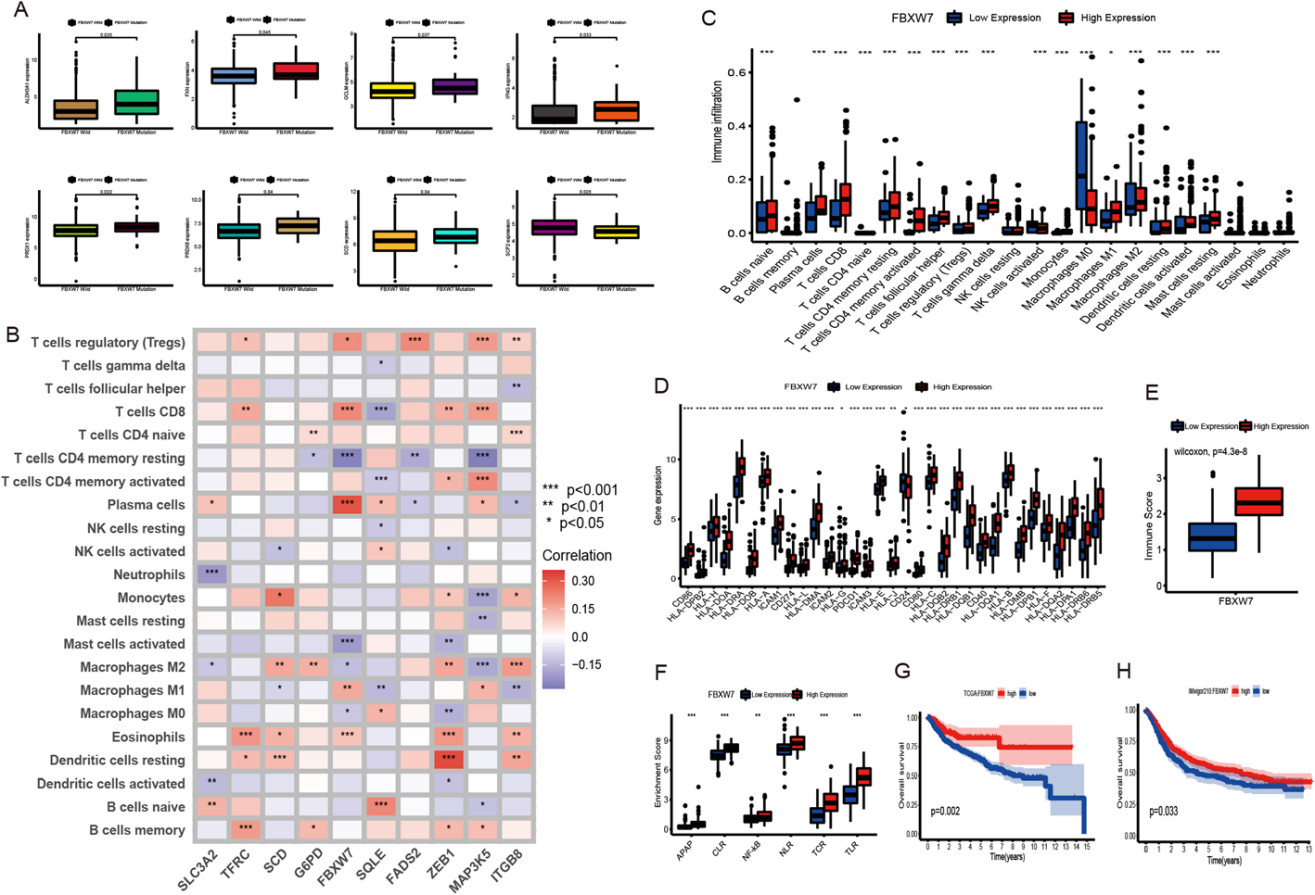
Correlation between TME infiltration cells and ferroptosis regulators and the roles of FBXW7 in activation of dendritic cells. **A** Difference in gene expression between FBXW7 mut and wt. **B** The correlation between TME infiltration cells and ferroptosis regulators. **C** Difference in the abundance of TME infiltrating cells. **D** Difference in expression of HLA genes and ICPs. **E** Difference in immuneScore. **F** Differences in immune-activated pathways. **G**, **H** Survival analyses for patients in TCGA (**G**) and anti-PD-L1 (**H**) immunotherapy cohort (*p < 0.05; **p < 0.01; ***p < 0.001)

Three GEO datasets (GSE13507, GSE32894 and GSE48075) and TCGA-BLCA cohort with available OS data and clinical information (Table 1) were enrolled into one meta-cohort. A univariate Cox regression model revealed the prognostic values of 55 FRGs in patients with bladder cancer (Additional file 1: Table S1). The comprehensive landscape of top 27 FRGs’ interactions, regulator connection and their prognostic significance for BCa patients were depicted with the FRG network (Additional file 1: Fig. S2A). We found that a significant correlation was shown among these FRGs. It was found that tumors with a high expression of (SLC3A2, TFRC, SCD, and G6PD) showed a low expression of ZEB1, while the high expression of SRC did not affect the expression of PRDX1 and RNF113A. Tumors with a high expression of FADS2, PRDX1, G6PD and TFRC showed a high expression of SLC38A1 and GCLM, GCLM and PGD also did not interfere with ZEB1 expression, while FBXW7 shared a common trend in gene expression with ZEB1. In addition, the change of SRC expression did not affect the expression of these two genes. Considering the relatively higher mutation frequency of writer gene FBXW7, we analyzed the difference in expression of other FRGs between FBXW7-mutant and wild types (Fig. 4A). Of these, SCD was significantly up-regulated in FBXW7- mutant tumors compared to wild-type tumors, while SCP2 was significantly down-regulated (Fig. 4A). The above results indicated that cross-talk among the regulators of different FRGs may play critical roles in the formation of different ferroptosis regulation patterns and TME cell-infiltrating characterization between individual tumors.

And we further examined the specific correlation between each TME infiltration cell type and each FRG using spearman’s correlation analyses (Fig. 4B). We focused on the regulator FBXW7, and revealed its significantly positive correlation with numerous TME infiltrating immune cells. We used ESTIMATE algorithm to quantify the overall infiltration of immune cells between high and low FBXW7 expression patients. The results showed that high expression of FBXW7 exhibited high immune scores, which meant that the TME with high expression of FBXW7 existed a significantly increased immune cell infiltration, thus confirming the above findings (Fig. 4E). We then explored the specific difference of 23 TME infiltrating immune cells between high and low FBXW7 expression patients. We found tumors with high expression of FBXW7 presented significantly increased infiltration in 23 TME immune cells compared to patients with high expression (Fig. 4C). Recent studies paid special attention to the mechanism of ferroptosis regulating the activation of dendritic cells (DCs). DCs, which are responsible for antigen presentation and the activation of naive T cells, are a bridge connecting innate and adaptive immunity, and their activation depending on the high expression level of MHC molecules, costimulatory factors and adhesion factors [28]. Our study indicated that tumors with high expression of FBXW7 showed significant more enrichment of TME DCs infiltration including activated DCs, immature DCs, and plasmacytoid DCs. We also noted that the decreased expression of FBXW7 resulted in the comprehensively elevated expression of MHC molecules, costimulatory molecules, and adhesion molecules (Fig. 4D). Subsequent pathway enrichment analyses, as expected, tumors with high FBXW7 expression exhibited an obvious enhancement in immune activation pathways including the pathway of antigen processing and presentation, C-type lectin receptor, NOD-like receptor, T cell receptor, Tolllike receptor and NF-κB signaling pathway (Fig. 4F). It was interesting that the immune-related pathway enhancements were accompanied by the increased expression of immunological checkpoint molecules PD1/L1 (Fig. 4D). So, we investigated whether the expression of FBXW7 regulator affected the therapeutic efficacy of immune checkpoint blockade. In meta-cohort and anti-PD-L1 immunotherapy cohort (IMvigor210), a survival benefit trend was observed in patients with high expression of FBXW7 (Fig. 4G, H). From above, we could speculate that FBXW7-mediated ferroptosis modification may promote the activation of TME DCs, thus enhancing the intratumoral anti-tumor immune response.

**Fig. 5:**
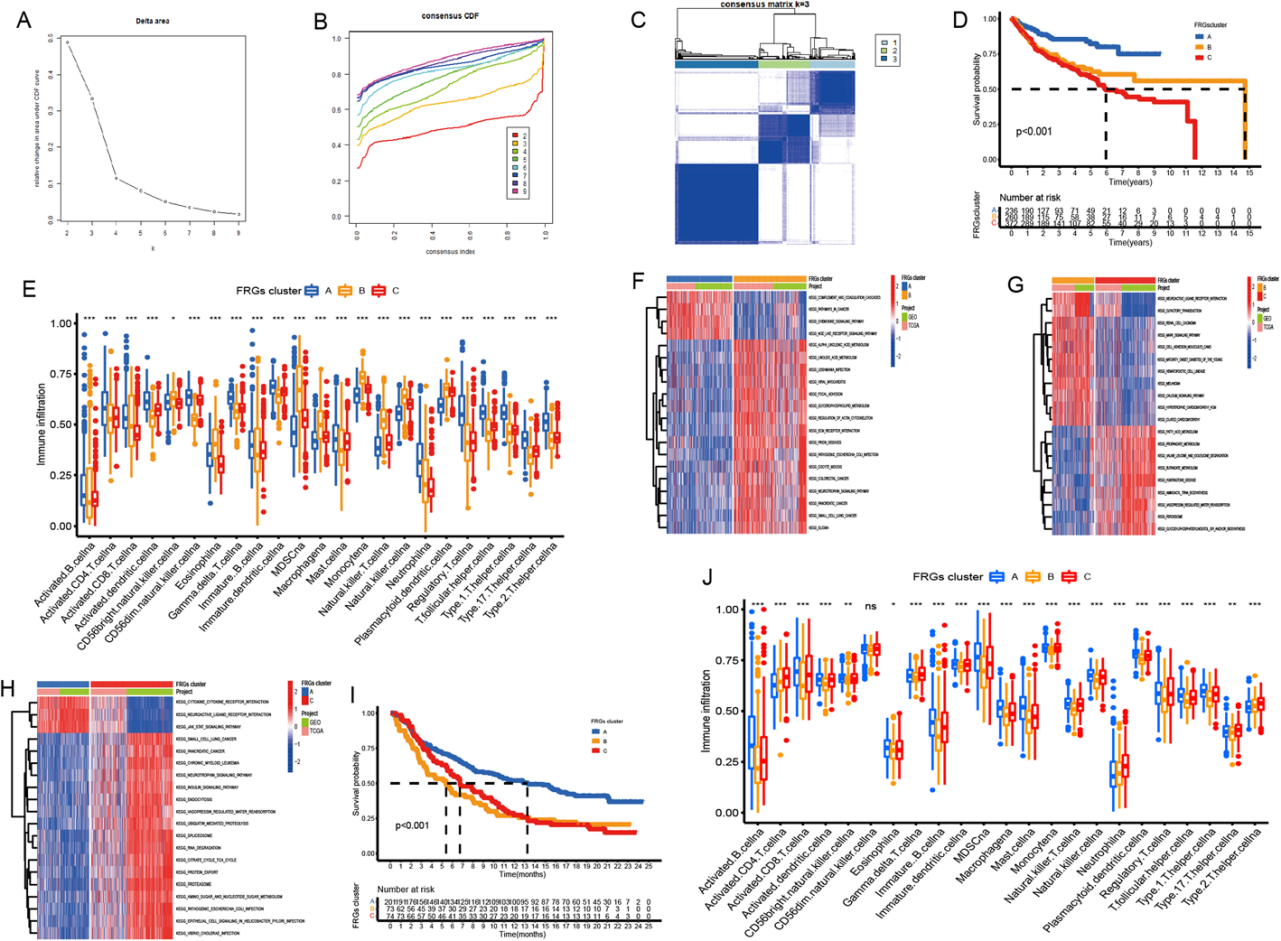
Patterns of ferroptosis regulation modification and biological characteristics of each pattern. **A**–**C** Unsupervised clustering of 55 ferroptosis regulators in a meta cohort (TCGA + GEO sets, n = 868, k = 3). **D** Survival analyses for the three ferroptosis regulation patterns based on 868 patients with BCa. **E** The abundance of TME infiltrating cells in three ferroptosis regulation patterns in meta cohort. **F**–**H** GSVA enrichment analysis. **I** Survival analyses in IMvigor210 cohort. **J** The abundance of TME infiltrating cells in three ferroptosis regulation patterns in IMvigor210 cohort

Interestingly, three distinct ferroptosis regulation patterns were eventually identified based on the expression of 55 FRGs using unsupervised clustering (Fig. 5A–C), including 236 cases in pattern A, 260 cases in pattern B and 372 cases in pattern C. We termed these patterns as FRGs cluster A–C, respectively (Fig. 5D and Additional file 1: Fig. S2C). Prognostic analysis for the three main ferroptosis subtypes revealed the particularly prominent survival advantage in FRGscluster-A regulation pattern (Fig. 5D).

To explore the biological behaviors among these distinct ferroptosis regulation patterns, we performed GSVA enrichment analysis. As shown in Fig. 5G, H, FRGscluster-A was markedly enriched in immune fully activation including the activation of chemokine signaling pathway, cytokine-cytokine receptor interaction and NOD like receptor signaling pathways. FRGscluster-B presented enrichment pathways associated with stromal and carcinogenic activation pathways such as ECM receptor interaction, JAK-STAT signaling pathway, cell adhesion. While FRGscluster-C was prominently related to immune and metabolism suppression biological process.

We then used the CIBERSORT method, a deconvolution algorithm using support vector regression for determining the immune cell type in tumors, to compare the component differences of immune cells among the three ferroptosis regulation patterns. To our surprise, subsequent analyses of TME cell infiltration indicated FRGscluster-A was remarkably rich in innate immune cell infiltration including activated B cell, activated CD4 T cell, activated CD8 T cell, activated dendritic cell, Gamma delta T cell, Immature B cell, Immature dendritic cell, Type 2 T helper cell and Type 17 T helper cell (Fig. 5E), and FRGscluster-B was remarkably rich in more immune cell infiltration such as eosinophil, MDSC, Macrophage, mast cell, natural killer cell, natural killer T cell, neutrophil cell, plasmacytoid dendritic cell and Regulatory T cell. Consistent with the abovementioned, patients with three ferroptosis pattern did show a matching survival difference (Fig. 5D). Previous studies demonstrated that tumors with immune excluded phenotype also showed the presence of abundant immune cells, while these immune cells were retained in the stroma surrounding tumor cell nests rather than penetrate their parenchyma. The activation of stroma in TME were considered T-cell suppressive [29]. The results from GSVA analyses have revealed cluster B regulation pattern was significantly associated with stromal activation. Therefore, we speculated that stromal activation in cluster B inhibited the anti-tumor effect of immune cells. Subsequent analyses showed that stroma activity was significantly enhanced in cluster B such as the activation of epithelial-mesenchymal transition (EMT), Janus Kinase (JAK) and angiogenesis pathways, which confirmed our speculation Based on the above analyses, we were surprised to find three ferroptosis regulation patterns had significantly distinct TME cell infiltration characterization. Cluster A was classified as immune-inflamed phenotype, characterized by adaptive immune cell infiltration and immune activation; cluster B was classified as immune-excluded phenotype, characterized by innate immune cell infiltration and stromal activation; cluster C was classified as immune-desert phenotype, characterized by the suppression of immunity (Fig. 5E–H).

### Validation of ferroptosis regulation patterns in IMvigor210 cohort

To further explore the characteristics of these ferroptosis phenotypes in the different clinical traits and biological behaviors, we fixed attention on the IMvigor210 cohort, which comprised 348 urothelial cancer patients and offered the most comprehensive clinical annotation. Similar to BCa meta-cohort clustering, unsupervised clustering also discovered three fully distinct patterns of ferroptosis in IMvigor210 cohort (Additional file 1: Fig. S3A–D). There was significant distinction existed on the ferroptosis transcriptional profile among three different ferroptosis regulation patterns (Additional file 1: Fig. S3E, F). FRGs cluster A was characterized by the increased expression of FADS2 and ALOX5, and presented variable decreases in other FRGs; FRGs cluster B showed high expression of CDO1, ABCC1, ALDH3A1, EIF4A1, EMP1, IFNG, TMBIM4, TGFBR1, LAMP2, NOX1, MYC and ACSL5; and FRGs cluster C exhibited significant increases in the expression of G6PD, TFRC, FXN and LOX. Patients with immune-inflammed subtype were characterized by the FRGs cluster-A regulation patterns, while immune-excluded were characterized by the FRGs cluster-C regulation patterns, and FRGs cluster-B regulation patterns belonged to immune-desert subtype (Fig. 5J). We also noted that tumors with FRGs cluster-A patterns presented better differentiation and were enriched in the TCGA subtype-I. In bladder cancer, the TCGA subtype with poorer differentiation was markedly linked to a poorer survival. Therefore, the tumors characterized by FRGs cluster-B regulation patterns were significantly correlated with stromal activation, high malignancy and rapid progression (Fig. 5J, Additional file 1: Fig. S3E). One-way ANOVA test also confirmed the remarkable differences on FRG expression between three key ferroptosis regulation patterns. Prognostic analysis also revealed FRGs cluster A to be markedly related to prolonged survival, while FRGs cluster B and FRGs cluster C were characterized by poorer survival (Fig. 5I).

### Generation of ferroptosis phenotype-related genes scores and functional annotation

To further investigate the potential biological behavior of each ferroptosis regulation pattern, we determined 3310 ferroptosis phenotype-related DEGs using limma package (Additional file 1: Fig. S4A, B). The “clusterProfiler” package was used to perform GO enrichment analysis for the DEGs. The biological processes with significant enrichment were summarized in Additional file 1: Fig. S4C, D. Surprisingly, these genes showed enrichment of biological processes remarkably related to ferroptosis and immune, which confirmed again that ferroptosis played a nonnegligible role in the immune regulation in tumor microenvironment.

To further validate this regulation mechanism, we then performed unsupervised clustering analyses based on the obtained 3310 ferroptosis phenotype-related genes in order to classify patients into different genomic subtypes. Consistent with the clustering grouping of ferroptosis regulation patterns, the unsupervised clustering algorithm also revealed three distinct ferroptosis genomic phenotypes and we named these three clusters as ferroptosis gene cluster A–C, respectively (Additional file 1: Figs. S4E–5F). This demonstrated that three distinct ferroptosis regulation patterns did exist in bladder cancer. Patients with alive status were mainly concentrated in the ferroptosis gene cluster A, while patients with clinical stage IV were characterized by the ferroptosis gene cluster C patterns. Analysis also indicated three distinct gene clusters were characterized by different signature genes (Additional file 1: Fig. S5A–D). 269 of 868 patients with bladder cancer were clustered in gene cluster A, which were proved to be related to better prognosis. While patients in gene cluster C (413 patients) experienced the outcome of poorer prognosis. An intermediate prognosis was observed in gene cluster B, with 186 patients clustered (Additional file 1: Fig. S4F). In the three FRG gene clusters, the prominent differences in the expression of FRGs were observed, which was in accordance with the expected results of ferroptosis regulation patterns (Additional file 1: Fig. S4G).

Considering the individual heterogeneity and complexity of ferroptosis, based on these phenotype-related genes, we constructed a set of scoring system to quantify the ferroptosis pattern of individual patients with bladder cancer, we termed as FRGs score. The Sankey diagram was used to visualize the attribute changes of individual patients (Additional file 1: Fig. S4I).

Next, we sought to further identify the value of FRGs score in predicting patients’ outcome. With the cutoff value -13 determined by survminer package, patients were divided into low or high FRGs score group. Patients with low FRGs score demonstrated a prominent survival benefit (HR 2.23 (1.68–3.89); Additional file 1: Fig. S4H), with 5-year survival rate significantly higher than patients with high FRGs score (82.5% vs 56.8%). We also found that FRGs score was distributed differently in the FRGs clusters and gene clusters, with the highest level in clusterC (Additional file 1: Fig. S4J). And the FRGs score is closely related to the level of immune-infiltrating cells in tumors. For example, the higher FRGs score is, the lower activated B cell, activated CD4 T cell and activated CD8 T cell infiltration is (Additional file 1: Fig. S4K).

### Identification of a brief lncRNA signature related to ferroptosis for prognosis and treatment

Pearson correlation analysis was applied to calculate the correlation between lncRNAs and ferroptosis-related genes. This analysis led to identification of 1329 lncRNAs associated with ferroptosis genes with a correlation coefficient > 0.4 and p < 0.01. After comparison of expression of genes between high and low FRG score, 607 lncRNAs were obtained by the Wilcoxon test. Then, 302 lncRNAs were finally obtained by the limma analysis between 19 normal-tissue samples and 406 BC samples. Last, the 406 BC samples were divided randomly into a “training” set (203 samples) and “testing” set (203 samples) for subsequent analyses.

**Fig. 6:**
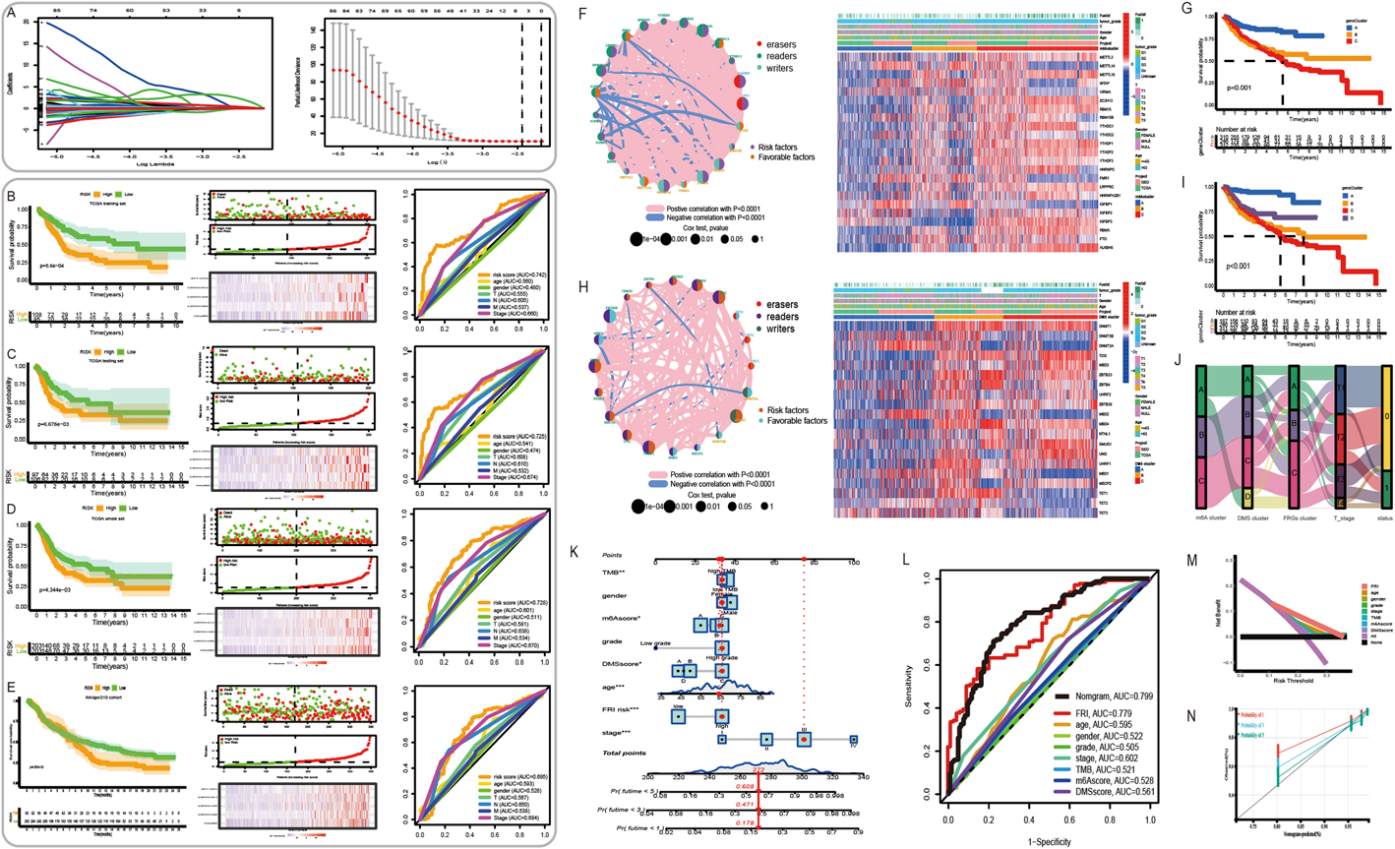
Establishment of a 5-lncRNA signature and a comprehensive nomogram. **A** Illustration for LASSO coefficient profiles of 44 prognostic lncRNAs. **B**–**E** The performance of a 5-lncRNA signature in Training group (**B**), testing group (**C**), TCGA cohort (**D**), IMvigor210 cohort (**E**). **F**, **H** The interaction between m6 A regulators (**F**) and DNA methylation regulators (**H**). **G**–**I** Kaplan–Meier analyses. **J** ggAlluvial diagram. **K** The comprehensive nomogram in TCGA dataset. **L** ROC analyses. **M**, **N** The DCA curve and calibration curve

Then, forty-eight prognostic lncRNAs (p < 0.001) were identified by univariate Cox survival analysis in the training group. Because the number of prognostic lncRNAs was too high, six key prognostic lncRNAs (LINC01426, LRP4-AS1, LINC01098, C6orf99, LINC01614, ST7-OT4) were selected through a least absolute shrinkage and selection operator (LASSO) regression model (Fig. 6A). Subsequently, we calculated the coefficient (β_i_) of 5 lncRNAs (ST7-OT4, LIINC01614, LINC01426, LINC01098, C6orf99) after multivariate Cox regression analysis (MCRA), and revealed the hazard ratio (HR) with 95% confidence interval (CI), respectively (Additional file 1: Table S2).

Based on the estimated regression coefficient of the five lncRNAs, a formula for the risk score was developed. The ferroptosis risk index (FRI) of each patient was calculated based on the following formula: FRI = (0.2654 × relative expression of LINC01426) + (1.1533 × relative expression of LINC01098) + (0.3617 × relative expression of C6orf99) + (0.0352 × relative expression of LINC01614) + (5.1652 × relative expression of ST7-OT4). A heatmap of the expression profile of the five lncRNAs and distribution of the risk score, survival status along with survival duration of BCa patients and relative expression of five key prognostic lncRNAs are shown in Fig. 6B–E. Among these lncRNAs, they were all risk factors positively correlated with OS (p < 0.0001). All BCa patients were stratified into “low-risk” and “high-risk” groups using the median value of the risk score as the cutoff. Overall, data indicated that a higher risk score predicted a shorter OS of BC patents in the training set, and this theory was verified in a validation set, IMvigor210 cohort.

Comparison of OS between patients with a high FRI and patients with a low FRI was undertaken using the median value of the FRI as the cutoff. Results suggested that patients with a higher FRI had shorter OS than patients with a low FRI in the training set (HR = 1.564, p < 0.001) (Fig. 6B, Additional file 1: Table S3), testing set (1.247, < 0.001) (Fig. 6C, Additional file 1: Table S3) and training set + testing set (1.048, < 0.001) (Fig. 6D, Additional file 1: Table S3). Analyses of ROC curves indicated that the five-lncRNA signature had “ideal” sensitivity and specificity for prognostic prediction of BCa patients, with all areas under the ROC curve (AUC) > 0.72 in the TCGA cohort (Fig. 6B–D). We also undertook analyses of survival and ROC curves in the IMvigor210 cohort (AUC = 0.685) (Fig. 6E). Analyses of Kaplan–Meier curves also indicated that patients with a high FRI carried a poor prognosis (p < 0.01) (Fig. 6B–E). Samples in the whole TCGA cohort were subjected to principal component analysis (PCA) based on the FRI of the 5-lncRNA signature, and were divided into high-risk and low-risk groups for OS of patients with BC. PCA showed that the 5-lncRNA signature predicted OS (Fig. 6B–E). Moreover, we conducted univariate Cox regression analysis in TCGA cohort to screen significant clinical features for the prognosis, during which the FRI, age, gender, stage, T stage, N stage, and M stage of the American Joint Committee on Cancer classification were included. Univariate analysis showed that the 5-lncRNA signature, age, T stage, N stage, M stage and stage were correlated significantly with OS (Additional file 1: Table S3). To ascertain whether the 5-lncRNA signature could be an independent prognostic factor for BC patients, MCRA was done using the FRI and other clinical features. The 5-lncRNA signature was a significant independent prognostic factor in the three cohorts (Additional file 1: Table S2). MCRA showed that only the 5-lncRNA signature, age, N stage, and M stage remained significantly associated with OS in the whole TCGA cohort (Additional file 1: Table S5).

### Cellular and molecular characteristics of two FRI subtypes for selecting the right patients for precise treatment

**Fig. 7:**
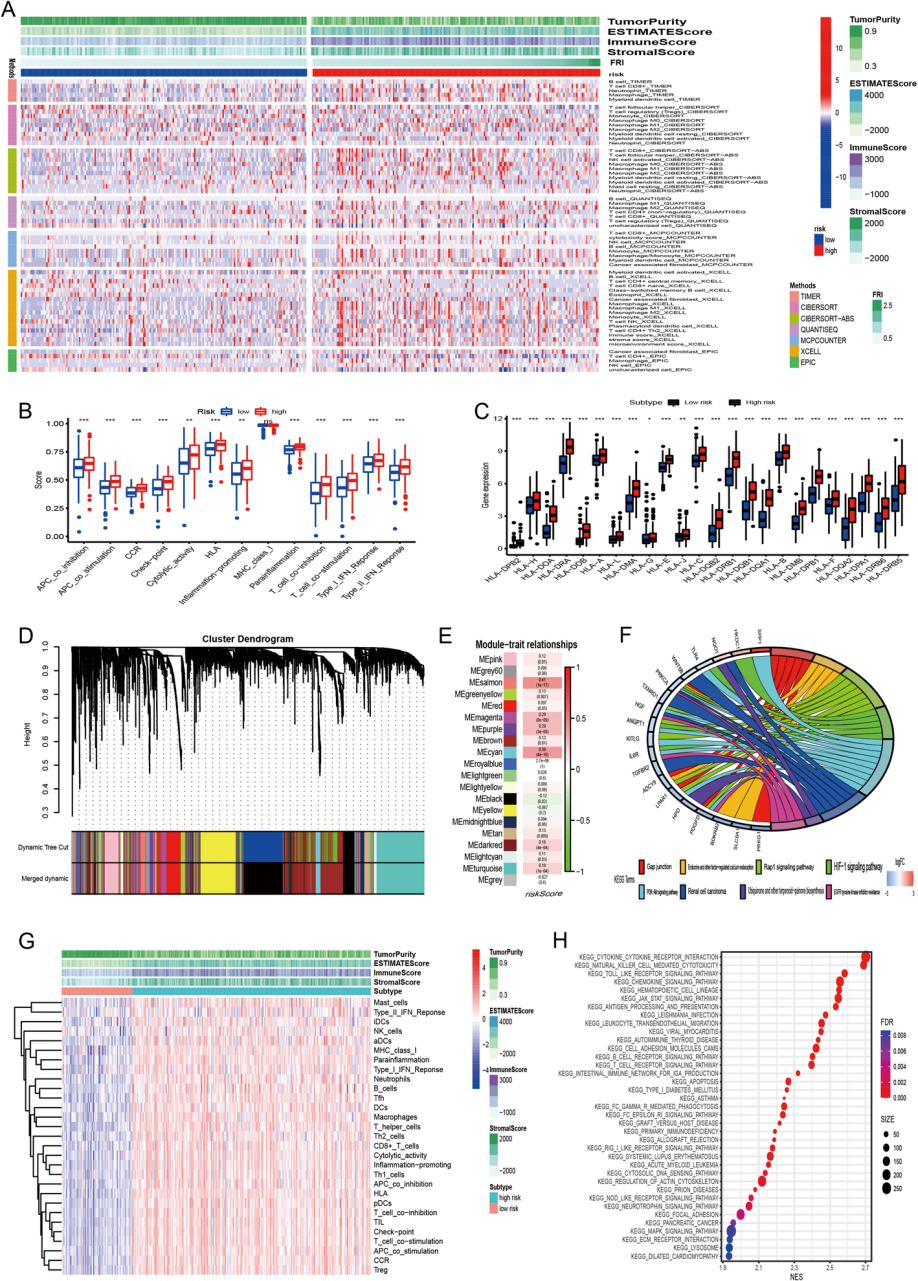
Cellular and molecular characteristics of 5-lncRNA risk subtypes. **A** The landscape of immune cell infiltration in TCGA cohort. **B** Difference in the immune related signaling pathways. **C** Gene expression of HLA gene sets between two distinct clusters. **D** Dendrogram of all differentially expressed genes clustered based on a dissimilarity measure (1-TOM). **E**, **F** Weight gene co-expression analysis and function enrichment analysis. **G** The landscape of immune cell infiltration in IMvigor210 cohort. **H** Functional annotation in IMvigor210 cohort

The response to mRNA vaccine depends on the tumor immune status. Hence, we further characterized the immune cell components in FRI subtypes by scoring 28 previously reported signature genes in both TCGA and IMvigor210 cohorts using ssGSEA. As shown in Fig. 7A, the immune cell components were divided into two clusters. The two clusters showed the different trend of immune cell infiltration. Further, the immune cell composition was significantly different among the subtypes. For instance, the immune scores were significantly higher in high-risk cluster compared to low-risk cluster (Additional file 1: Fig. S6A), and that of eosinophils, activated CD8 T cells, activated B cells, monocytes and effector memory CD4 T cells were higher in high-risk cluster relative to low-risk cluster. Thus, high risk cluster are immunological “hot” while low risk cluster are immunological “cold” phenotypes. Similar trends were seen in IMvigor210 cohort as well (Fig. 7G, Additional file 1: Fig. S7A). These results suggest that FRI subtype reflects the BCa immune status, and can identify suitable patients for mRNA vaccination. The mRNA vaccine with these antigens can induce immune infiltration in patients with immunologically “cold” low risk cluster tumors.

For example, low risk cluster showed lower counts of activated B cells, activated CD4+ T cells, activated CD8+ T cells, effector memory CD8+ T cells, regulatory T cells and myeloid-derived suppressor cells (MDSCs), while high risk cluster scored higher in terms of activated B cells, activated CD8+ T cells, effector memory CD8+ T cells, regulatory T cells and MDSCs. Thus, the mRNA vaccine may be relatively viable and more effective in low-FRI than in high-FRI group.

### WGCNA analysis and identification of biological pathways associated with the 5-lncRNA signature

The 5-lncRNA signature had a strong discriminatory power for the prognosis of BCa patients, so this signature might be closely associated with the biological pathways of BCa. To further explore the key mechanisms of the 5-lncRNA, we clustered the hub genes that were highly correlated with the FRI by weighted gene co-expression network analysis (WGCNA) in TCGA cohort. 20 modules were obtained after merging dynamic and clustering analysis, and only the cyan and salmon module was correlated significantly with the FRI (cyan: correlation = 0.39, p = 4 × 10^–16^; salmon: correlation = 0.41, p = 1 × 10^–17^) (Fig. 7D, E, Additional file 1: Fig. S6B).

Based on the genes included in the cyan and salmon module, we tried to elucidate the underlying biological pathways using the Gene Ontology (GO) and Kyoto Encyclopedia of Genes and Genomes (KEGG) databases. Three signaling pathways [Rap1, hypoxia-inducible factor (HIF), phosphoinositide 3-kinase/protein kinase B (PI3K/AKT)] were the primary ones which the 5-lncRNA appeared to be involved in. Other ferroptosis-associated processes (e.g., regulation of extent of cell growth, positive regulation of JNK cascade and cartilage development) were also enriched significantly (Fig. 7F and Additional file 1: Fig. S6C). And we also found that patients in FRI-high group enriched in immune related pathways (Fig. 7H).

### Association between FRI subtypes of BCa and immune modulators

We identified that the higher FRI resulted in the comprehensively elevated expression of MHC molecules, costimulatory molecules, and adhesion molecules (Fig. 7B). Subsequent analyses of relationship between HLA gene sets and two FRI subtypes, as expected, the patients in FRI-high group have significantly higher expression than patients in FRI-low group (Fig. 7C). And the same goes for IMvigor210 cohort (Additional file 1: Fig. S7B).

Given the importance of immune checkpoints (ICPs) and immunogenic cell death (ICD) modulators in cancer immunity, we next analyzed their expression levels in the different FRI subtypes. Forty-seven ICPs related genes were detected in both cohorts, of which 41 (87%) genes in TCGA cohort (Additional file 1: Fig. S8A–E) and 46 (97.9%) in IMvigor210 cohort (Additional file 1: Fig. S8G–J) were differentially expressed between the high and low-FRI subtypes. For instance, ADORA2A, BTLA, CD160, CD27, CD40LG, CD48, CTLA4, ICOS, ICOSLG, IDO2, LAG3, LAIR1,NRP1, PDCD1, PDCD1LG2, TIGIT, TNFRSF14, TNFRSF25, TNFRSF4, TNFRSF8, TNFSF14, TNFSF15, TNFSF18, and VSIR were significantly upregulated in high-FRI tumors in the IMvigor210 cohort, and ADORA2A, BTLA, CD200, CD200R1, CD244, CD27, CD28, CD40, CD40LG, CD48, CD80, CD86, CTLA4, HAVC R2, ICOS, IDO1, IDO2, LAG3, LAIR1, PDCD1, PDCD1LG2, TIGIT, TMIGD2, TNFRSF18, TNFR SF25, TNFRSF4, TNFRSF8, TNFRSF9, and VSIR were also overexpressed in high-FRI tumors in TCGA cohort.

Furthermore, the overall expression level of ICPs in the IMvigor210 cohort was higher than that in TCGA cohort. Twenty-five ICD genes were detected in the IMvigor210 cohort, of which 18 (72%) were differentially expressed among the FRI subtypes (Additional file 1: Fig. S8K). Likewise, twenty-five ICD genes were expressed in TCGA cohort, of which 19 (76%) showed significant differences between the subtypes (Additional file 1: Fig. S8F). For instance, CALR, CXCL10, IFNAR1, MET, EIF2A and PANX1 were significantly upregulated in low-FRI tumors in TCGA cohort, while CALR, MET, TLR4, LRP1, EIF2A, ANXA1 and P2RY2 showed significantly higher expression levels in low-FRI tumors in IMvigor210 cohort. Therefore, FRI can reflect the expression levels of ICPs and ICD modulators and be treated as potential therapeutic biomarkers for mRNA vaccines.

### Relationship between FRI and tumor somatic mutation, microsatellite instability

**Fig. 8:**
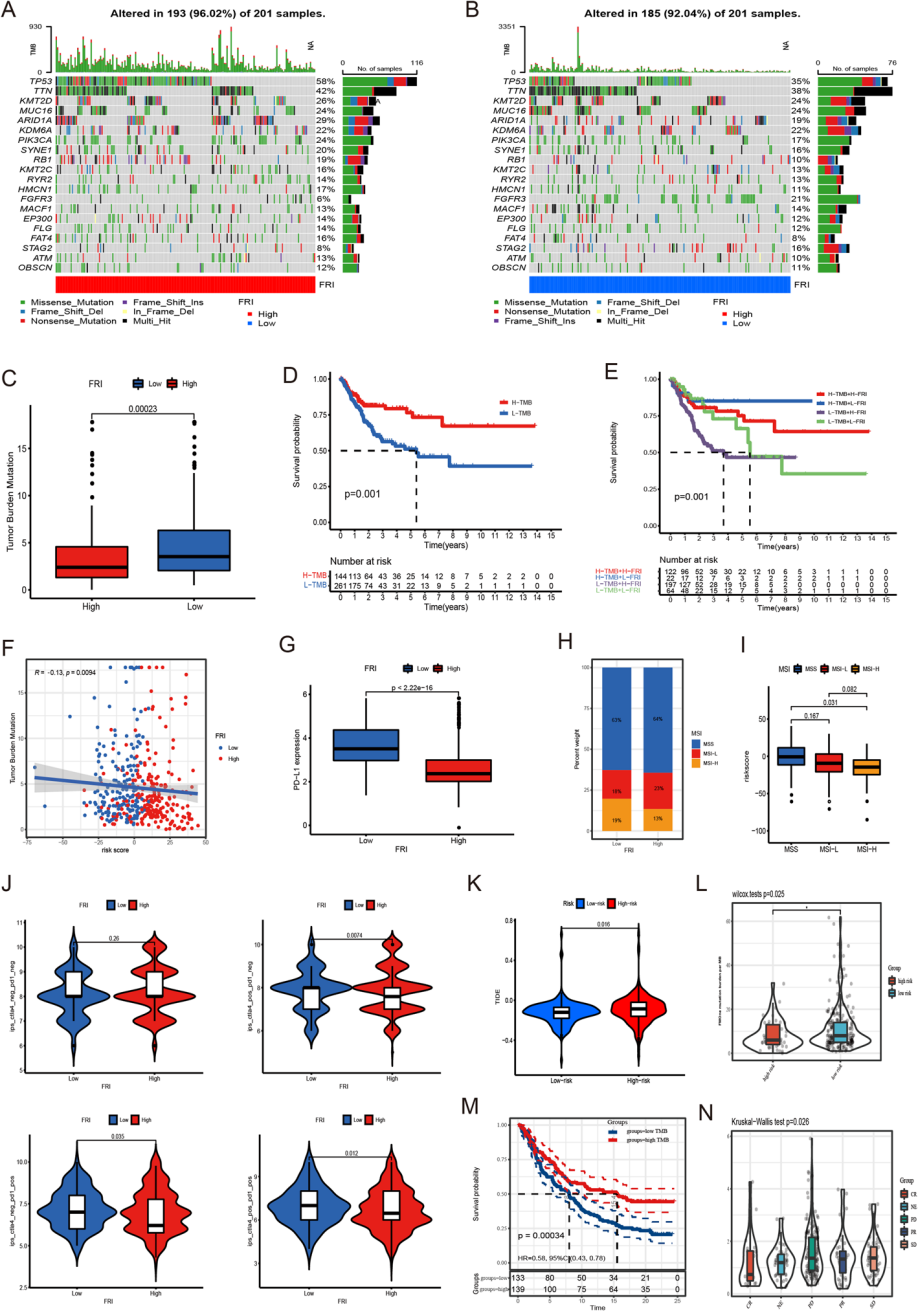
Characteristics of 5-lncRNA signature in TCGA molecular subtypes and relationship with tumor somatic mutation and MSI. **A**, **B** The waterfall plot of tumor somatic mutation. **C** Difference of TMB. **D** Survival analyses in TCGA cohort. **E** Survival analyses stratified by both FRI and TMB. **F** Correlation between FRI level and TMB in TCGA cohort. **G** Difference in PD-L1 expression. **H** The proportion of MSI subtype. **I** Differences of FRI among three MSI status in TCGA cohort. **J** The association between IPS and FRI. **K** Difference of TIDE score. **L** Differences of TMB in IMvigor210 cohort. **M** Survival analyses by median TMB in IMvigor210 cohort. **N** Differences of FRI among distinct immunotherapy response phenotypes in IMvigor210 cohort

Then, we analyzed the distribution differences of somatic mutation between FRI-low and FRI-high subgroup in TCGA-BLCA cohort using “maftools” package. As shown in Fig. 8A, B, FRI-low group presented more extensive tumor mutation burden than the high-risk score group. The TMB quantification analyses confirmed the low-risk score tumors was markedly correlated with a higher TMB (Fig. 8C). The FRI and TMB also exhibited a significant negative correlation (Fig. 8F, Additional file 1: Fig. S11A). Accumulated evidence demonstrated patients with high TMB status presented a durable clinical response to anti-PD-L1 immunotherapy. Therefore, the above results indirectly demonstrated that the difference in tumor ferroptosis regulation patterns could be a crucial factor that mediated the clinical response to anti-PD-L1 immunotherapy. And the values of FRI in predicting immunotherapeutic outcomes were also indirectly confirmed.

The clinical trials as well as preclinical researches have revealed patients with higher somatic TMB were correlated with enhanced response, long-term survival and durable clinical benefit when treated with immune checkpoint blockade therapy. The individual altered genes could mediate resistance or sensitivity to immunotherapy. For specific altered genes in TCGA-BLCA such as TP53, TTN, ARID1A, PIK3CA, SYNE1 and RB1, mutant type had significantly higher FRI compared to wild type, as well as FGFR3 and STAG2, mutant type had significantly lower FRI compared to wild type (Fig. 8A, B, Additional file 1: Fig. S9A, B). These results would provide novel perspective for exploring the mechanisms of ferroptosis modification in the tumor somatic mutations, shaping of TME landing, and roles in immune checkpoint blockade therapy.

The highly microsatellite instability subtype, characterized by better prognosis, was significantly correlated with lower FRI, whereas MSI-Low and MSS had a higher FRI (Fig. 8H, I). Multivariate analysis for TCGA-BLCA cohort also confirmed that FRI could act as an independent prognostic biomarker in bladder cancer (Additional file 1: Table S5). Previous studies indicated that patients with higher MSI or higher TMB in bladder cancer have been shown to respond to anti-PD-L1 antibodies in several studies. In our study, higher MSI or higher TMB in patients were markedly associated with lower FRI, and we found patients with combination of low-FRI and high mutation burden showed a great survival advantage (Fig. 8D, E), which implied FRI could be a more effective biomarker for the prediction of immunotherapeutic efficacy than MSI and TMB in patients with bladder cancer (Fig. 8E, I).

In addition, the immunogenicity of FRI was evaluated by IPS analysis. The IPS, IPS-CTLA4, IPS-PD1 and IPS-PD1-CTLA4 scores were higher in the FRI-low group (Fig. 8J). To evaluate the ability of FRI served as a biomarker predictive of the clinical response to ICB therapy. We analyzed datasets from patients with stage III/IV ccRCC using the TIDE algorithm. And the results showed that the FRI-low group was associated with lower TIDE scores, indicating a stronger response to ICB therapy (Fig. 8K).

### Analyzing the role of FRI in anti-PD-L1 immunotherapy

In anti-PD-L1 cohort (IMvigor210), patients with low FRI exhibited significantly clinical benefits and a markedly prolonged survival [Fig. 6E; IMvigor210, HR 1.623 (1.203–1.996), Additional file 1: Table S3]. The significant therapeutic advantages and clinical response to anti-PD-L1 immunotherapy in patients with low-risk score compared to those with high-risk score were confirmed (Fig. 8L, N and Additional file 1: Fig. S10B). In addition, patients with low-risk score showed an obviously high expression of PD-L1, which indicated a potential response to anti-PD-L1 immunotherapy (Fig. 8G). Further research revealed that regulatory T-cells and TME stroma were significantly activated in tumors with high-risk score, which mediated immune tolerance of tumors (Fig. 7G).

The above implied that the quantification of ferroptosis regulation patterns was a potential and robust biomarker for prognosis and clinical response assessment of immunotherapy (Fig. 8L, N, Additional file 1: Fig. S10A, C). The immune phenotypes of tumors in the IMvigor210 cohort have been detected, so we investigated the difference of risk score among different phenotypes. We found that higher risk score was remarkably associated with exclusion and desert immune phenotypes, and checkpoint inhibitors were difficult to exert anti-tumor effect in these phenotypes (Additional file 1: Fig. S10C). In summary, our work strongly indicated that ferroptosis regulation patterns was significantly correlated with tumor immune phenotypes and response to anti-PD-L1 immunotherapy, and the established ferroptosis signature would contribute to predicting the response to anti-PD-L1 immunotherapy. These findings suggest that FRI can predict TMB and somatic mutation rates in BCa patients, and that patients with low risk may respond positively to anti-PD-L1 immunotherapy.

In addition, we revealed that elderly patients, diffuse histological subtype and advanced patients were significantly associated with a higher FRI, which meant that these patients were characterized with a poorer clinical outcome. These results demonstrated FRI could be also used to evaluate certain clinical characteristics of patients such as MSI status, molecular subtypes, histological subtypes as well as clinical stage, etc. (Additional file 1: Fig. S11A–C).

### Identify the value of m6A and DNA methylation score (DMS) cluster in predicting patients’ outcome

m6A score and DMS was constructed to quantify ferroptosis regulation patterns of individual tumors using principal component analysis algorithms, just like calculating FRGs score (Fig. 6F–J).

### Construction of nomograms and validation

According to MCRA results, a comprehensive nomogram was generated for “individualized” prediction of OS at 1 year, 3 years and 5 years that integrated independent prognostic features (age, T stage, N stage, M stage, m6A score, DMS score, FRI) (Fig. 6K). For this nomogram, AUC ≤ 0.799, so it could be employed to discriminate between patients with a poor prognosis from patients with a favorable prognosis (Fig. 6L). Besides, The DCA curve and calibration curve indicated that a nomogram was feasible to make valuable and profitable judgments (Fig. 6M).

## Discussion

Increasing evidence demonstrated that ferroptosis took on an indispensable role in inflammation, innate immunity as well as anti-tumor effect through interaction with various ferroptosis related regulators. As most studies focus on single TME cell type or single regulator, the overall TME infiltration characterizations mediated by integrated roles of multiple FRGs are not comprehensively recognized. Identifying the role of distinct ferroptosis regulation patterns in the TME cell infiltration will contribute to enhancing our understanding of TME anti-tumor immune response, and guiding more effective immunotherapy strategies.

Here, based on 55 FRGs, we revealed three distinct ferroptosis regulation patterns. These three patterns had significantly distinct TME cell infiltration characterization. Cluster A was characterized by the activation of adaptive immunity, corresponding to immune-inflamed phenotype; cluster B was characterized by the activation of innate immunity and stroma, corresponding to immune-excluded phenotype; cluster C was characterized by the suppression of immunity, corresponding to immune-desert phenotype. The immune-excluded and immune-desert phenotypes could be regarded as non-inflamed tumors. The immune-inflamed phenotype, known as hot tumor, show by a large number of immune cell infiltration in TME [29–31]. Although the immune-excluded phenotype also showed the presence of abundant immune cells, the immune cells were retained in the stroma surrounding tumor cell nests rather than penetrate their parenchyma. The stroma could be confined to the tumor envelope or may penetrate the tumor itself, making the immune cells appear to be really inside the tumor [32–34]. The immune-desert phenotypes were associated with immune tolerance and ignorance, and lack of activated and priming T-cell [35]. Consistent with the above definitions, we found cluster B exhibited a significant stroma activation status, including the highly expressed angiogenesis, EMT and TGF-β pathways, which were considered T-cell suppressive. Combined with the TME cell-infiltrating characteristics in each cluster, it confirmed the reliability of our classification of immune phenotypes for different ferroptosis regulation patterns. Therefore, after fully exploring the TME cell-infiltrating characterization induced by distinct ferroptosis regulation patterns, it was not surprising that cluster B had the activated innate immunity but poorer prognosis than cluster A.

Further, in this study, the mRNA transcriptome differences between distinct ferroptosis regulation patterns have been proved to be significantly associated with ferroptosis and immune related biological pathways. Similar to the clustering results of the ferroptosis phenotypes, three genomic subtypes were identified based on ferroptosis phenotype related genes, which were also significantly correlated with stromal and immune activation. This demonstrated again that the ferroptosis was of great significance in shaping different TME landscapes. Therefore, a comprehensive assessment of the ferroptosis regulation patterns will enhance our understanding of TME cell-infiltrating characterization. Considering the individual heterogeneity of ferroptosis, it was urgently demanded to quantify the ferroptosis regulation patterns of individual tumor. For that, we established a set of scoring system to evaluate the ferroptosis pattern of individual patients with bladder cancer—FRGs score. The ferroptosis pattern characterized by immune-excluded phenotype exhibited a higher FRGs score, while the pattern characterized by immune-inflamed phenotype showed a lower FRGs score.

On the other hand, the component of FRGs score contains thousands of genes, which has limitations in practical application. Therefore, it is extremely necessary to establish a brief and representative model: FRI.

Thus, 5 Fr-lncRNAs were identified to predict the prognosis and tumor immune reaction of BCa for the first time. KM plot analysis, ROC curve analysis, random sampling validation, and subgroup analysis were performed for model verification and showed that the novel signature is a powerful tool for BCa prognosis prediction. Moreover, it was found that the risk score calculated using the Fr-lncRNA signature was significantly correlated with immune infiltration and immunotherapeutic effectiveness. GSEA analysis showed that the constructed risk model is ferroptosis related. In addition, In IMvigor210 cohort with the determined lncRNA signature phenotype, these results were well validated [36]. This suggested risk score was a reliable and robust tool for comprehensive assessment of individual tumor ferroptosis regulation patterns, which could be used to further determine the TME infiltration patterns, that was, tumor immune phenotypes. Integrated analyses also demonstrated that risk score was an independent prognostic biomarker in bladder cancer. The result showed that patients with low-risk score have better OS.

The Fr-lncRNA signature is an effective and practical tool for predicting the prognosis of patients with BCa. Compared with other clinical features, the novel model was able to distinguish cases with a high or low risk with higher efficacy. Univariate and multivariate analyses revealed that the risk score was an independent prognostic predictor. Random resampling verification, clinical parameter association analysis, and subgroup analysis validated the robustness of the model.

Among the lncRNAs in the Fr-lncRNA signature, several have been demonstrated to be related to immunity, ferroptosis, and malignancy. For instance, LINC01614, which was reported to suppress the malignant phenotypes of gastric cancer cells [37], could also regulate lung cancer cell proliferation and migration [38]. LINC01426 contributes to clear cell renal cell carcinoma progression by modulating CTBP1/miR-423-5p/FOXM1 axis via interacting with IGF2BP1 [39], and to lung cancer progression via AZGP1 and predicts poor prognosis in patients with LUAD [40]. LncRNAs(C6orf99) was identified that it can predict survival and tumor microenvironment characteristics in breast cancer [41, 42]. Overall, our model helped in identifying new biomarkers and in proposing novel mechanisms for BCa.

To the best of our knowledge, this is the first study to screen for BCa antigens for developing ferroptosis related mRNA vaccine. We constructed the aberrantly expressed and mutational landscape of BC and identified a series of targetable antigens, of which TFRC, SCD, G6PD, FADS2, SQLE, and SLC3A2 are promising mRNA vaccine candidates. Their upregulation was not only associated with poor prognosis and DFS, but also high APC and B cell infiltration. Therefore, these antigens play critical roles in the development and progression of BCa and can be directly processed and presented to CD8+ T cells in the event of adequate lymphocyte infiltration to induce an immune attack. Although these candidates have to be functionally validated, their potential for mRNA development is supported by previous reports. For example, A missense mutation in TFRC, encoding transferrin receptor 1, causes combined immunodeficiency [43] and TFRC also promotes epithelial ovarian cancer cell proliferation and metastasis via up-regulation of AXIN2 expression [44]. Suppressing SCD expression will inhibit esophageal cancer progression via activating the GADD45B-MAP2K3-p38-p53 feedback loop [45]. Lineage-Restricted Regulation of SCD and Fatty Acid Saturation by MITF Controls Melanoma Phenotypic Plasticity [46]. G6PD acts as an oncogene in many types of cancers and Nrf2 promotes breast cancer cell migration via up-regulation of G6PD/HIF-1α/Notch1 axis [47]. FADS2 knockout suppresses cancer cell proliferation, migration and invasion by inducing p53/p21-mediated G0/G1 phase blockade [48]. The aberrantly low levels and activation of FADS2 are closely related with cancer onset, progression and metastasis [39] through inhibiting Ferroptosis [49]. It shows that SQLE reduction caused by cholesterol accumulation, which correlated with tumor biological processes, especially oncogenic signaling pathways, ferroptosis, and tumor microenvironment, aggravates CRC progression via the activation of the β-catenin oncogenic pathway and deactivation of the p53 tumor suppressor pathway [50]. Despite the proven tumorigenic role of SLC3A2 in a number of cancers including head and neck squamous cell carcinomas (HNSCC), it is also a novel therapeutic approach for advanced prostate cancer [51].

Given that mRNA vaccine is only beneficial for a fraction of cancer patients, we classified BCa into high and low risk subtypes based on Fr-lncRNAs expression profiles for selecting the appropriate population for vaccination. The two risk score subtypes exhibited distinct molecular, cellular and clinical characteristics. Patients with low-risk score showed better prognosis compared to high-risk subtypes in both IMvigor210 cohort and TCGA cohorts. This suggests the ferroptosis related lncRNA signature can be used for predicting the prognoses of BCa patients, and we demonstrated its superior predictive accuracy compared to traditional staging and grading. In addition to prognostic prediction, Fr-lncRNA signature is also indicative of the therapeutic response to mRNA vaccine. For instance, patients with low-risk score with higher TMB may have greater responsiveness to mRNA vaccine. The high expressions of ICPs in high risk in IMvigor210 cohort cohort, and in high risk in TCGA cohort suggest an immunosuppressive tumor microenvironment, which may inhibit the mRNA vaccine from eliciting an effective immune response. In contrast, the elevated expression of ICD modulators in low risk in IMvigor210 cohort, and in low risk in TCGA cohort are suggestive of greater potential of mRNA vaccine in these immune subtypes. Interestingly, a missense mutation in TFRC, encoding transferrin receptor 1, causes combined immunodeficiency. And may therefore be a stronger candidate for mRNA vaccine compared to the other selected antigens. Since the tumor immune status is a determinant of mRNA vaccine efficacy, we further characterized the immune cell components in the different subtypes. High risk group showed significantly elevated scores of eosinophils, activated CD8 T cells, activated B cells, monocytes and effector memory CD4 T cells compared to low-risk group. This indicated that low risk group are immunological “cold”, and high-risk group are immunological “hot” phenotypes. The molecular signatures of these tumors were consistent with the immune signatures, indicating that patients with different immune subtypes respond distinctly to mRNA vaccine.

For instance, low risk was associated with low expression of CD8+ T cells, lymphocyte and stromal fraction and TGF-β response gene signatures, indicating an immunologically cold phenotype. To circumvent poor immunogenicity of these tumors, mRNA vaccines that stimulate the immune system by triggering immune cell infiltrating may be a suitable option. Combining the vaccine with ICB or ICD modulators, low risk group patients may reinvigorate the immune system and increase immune cell infiltration. High risk group exhibited a more immune-activated phenotype with low stromal fraction and TGF-β response gene expression, and may therefore be responsive to ICB and other strategies. Based on above ferroptosis-typing studies, BCa was classified into the A-C subtypes. A is associated with superior, B with moderate, and C with inferior prognoses. In this study, BC was differentiated into low risk-high risk subtypes. High risk mainly overlapped with C, low risk with A. These results were in agreement with better survival probability of low risk, and the relatively poor prognoses of High risk. Therefore, our risk-typing method is reliable and complements the previous classification. Nevertheless, the vaccine antigens and other prognostic markers identified in this study will have to be validated in future studies.

Our data also revealed a markedly negative correlation between risk score and tumor mutation burden. Consistent with previous studies, ferroptosis molecular subtypes demonstrated the lowest FRGs score, underlining the core role of immune activation in resistance to checkpoint immunotherapy [52]. This indicated that response to checkpoint immunotherapy was not only associated with antigen processing, and improved cytolytic activity, but also related to suppression of angiogenesis, fibroblast activation, TGF beta pathway components and the EMT. Previous studies confirmed that the EMT- and TGFbeta-related pathway activation resulted in decreased trafficking of T-cell into tumors as well as their weakened tumor killing effects [14, 52]. The above suggested that the activated stromal TME in the activated immune TME could mediate therapeutic resistance to immune-checkpoint blockade, as well as influence the individual precise immunotherapy of bladder cancer.

In this work, we showed ferroptosis regulation patterns played a nonnegligible role in shaping different stromal and immune TME landscape, implying ferroptosis could affect the therapeutic efficacy of immune checkpoint blockade. The Fr-lncRNA signature risk score with integrated various biomarkers including mutation load, PD-L1 expression and immune TME and MSI status, could be the more effective predictive strategy for immunotherapy. We also confirmed the predictive value of the risk score in one cohort with anti-PD-L1 immunotherapy. A significantly difference on risk scores existed between non-responders and responders.

Shortly, in clinical practice, the FR-lncRNA signature risk score could be used to comprehensively evaluate the ferroptosis regulation patterns as well as their corresponding TME cell infiltration characterization within individual patient, further to determine the immune phenotypes of tumors and guide the more effective clinical practice. We also demonstrated the risk score could be utilized for assessing patients’ clinicopathological features including stages of tumor inflammation, tumor differentiation levels, clinical stages, molecular subtypes, genetic variation, MSI status and tumor mutation burden etc. The detailed relationships between risk score and clinicopathological features could be found in our study. Similarly, risk score could act as an independent prognostic biomarker for predicting patients’ survival. We could also predict the efficacy of the patients’ clinical response to antiPD-L1 immunotherapy through risk score. More importantly, this study has yielded several novel insights for cancer immunotherapy that targeting FRGs or ferroptosis phenotype-related genes for changing the ferroptosis regulation patterns, and further reversing the adverse TME cell infiltration characterization, that was the transformation of “cold tumors” into “hot tumors”, may contribute to exploiting the development of novel drug combination strategies or novel immunotherapeutic agents in the future. Our findings provided novel ideas for improving the patients’ clinical response to immunotherapy, identifying different tumor immune phenotypes and promoting personalized cancer immunotherapy in the future, last for selecting appropriate patients for mRNA vaccine therapy.

## Conclusions

In conclusion, the difference of ferroptosis regulation patterns was a factor that could not be ignored to cause the heterogeneity and complexity of individual tumor microenvironment. And six ferroptosis related regulators (TFRC, SCD, G6PD, FADS2, SQLE, and SLC3A2) are potential BC antigens for mRNA vaccine development. Furthermore, we developed a novel lncRNA signature to help clinicians estimate the immunotherapy response, develop anti-BCa mRNA vaccine and selecting patients for vaccination.

## Supplementary Information

**Additional file 1**: **Table S1.** Univariate association of the 55-FrlncRNAs with overall survival in the meta-cohort. **Table S2.** Multivariate cox regression analysis of the 5-FrlncRNAs with overall survival in the TCGA train cohort. **Table S3.** Univariate association of the 5-lncRNAs ferroptosis-related signature with overall survival in the three sets. **Table S4.** Multivariate Cox regression analysis of the 5-lncRNAs-ferroptosis-related signature with overall survival in the three sets. **Table S5.** Univariate and Multivariate Cox regression analysis of the conserved 5-lncRNAs-ferroptosis-related signature with overall survival in TCGA cohort. **Figure S1.** Identification of tumor antigens associated with BCa prognosis. (A) Kaplan–Meier curves showing OS and DFS of BCa patients stratified on the basis of FADS2, SLC3A2, SCD, TFRC, SQLE,and G6PD expression levels. (B) Difference of mRNA expression level between four CNV types in TCGA cohort. (C) Kaplan–Meier curves showing OS of BCa patients stratified on the basis of FADS2, SLC3A2, SCD, TFRC, SQLE,and G6PD expression levels in meta cohort. **Figure S2.** (A) The interaction between ferroptosis regulators in bladder cancer. The circle size represented the effect of each regulator on the prognosis, and the range of values calculated by Log-rank test was p < 0.001, p < 0.01, p < 0.05 and p < 0.1, respectively. (B) The mutation co-occurrence and exclusion analyses between FBXW7 and other ferroptosis regulators. Co-occurrence, green; Exclusion, yellow. (C) The heatmap of unsupervised clustering of 55 ferroptosis regulators in the combined bladder cancer cohorts. The FRGs cluster, tumor stage, survival status and age were used as patient annotations. Red represented high expression of regulators and blue represented low expression. **Figure S3.** (A-D) Consensus matrices of the IMvigor210 cohort for k = 2—5. (E) Unsupervised clustering of 55 ferroptosis regulators in the IMvigor210 bladder cancer cohort. The FRGs cluster, immune phenotype, overall response, tumor stage, survival status and age were used as patient annotations. Red represented high expression of regulators and blue represented low expression. (F) Principal component analysis for the transcriptome profiles of three ferroptosis regulation patterns, showing a remarkable difference on transcriptome between different regulation patterns**. Figure S4.** Construction of ferroptosis related genes (FRGs) score. (A) Principal component analysis between different regulation patterns. (B) 3310 ferroptosis phenotype-related genes shown in venn diagram. (C-D) Functional annotation using GO and KEGG enrichment analysis. (E) Consensus matrices of the combined cohort for k = 3. (F) Survival analyses for the three ferroptosis phenotype-related gene cluster. (G) The expression of 55 ferroptosis regulators in three gene cluster. (H) Survival analyses of FRGs score (I) ggalluvial diagram plot. (J) Correlations between FRGs score and the known gene signatures. (K) Differences of FRGs score among different clusters. (p < 0.001). **Figure S5.** Characteristics of cytokine transcriptome, chemokine transcriptome and known signatures in distinct gene clusters (A) Difference in the immune-activation related gene expression among three gene clusters. (B) Difference in the immune-checkpoint related gene expression among three gene clusters. (C) Difference in the TGFβ- EMT pathway-related gene expression among three gene clusters. (D) Difference in the expression of known signatures including stromal-activation related signatures, tumor-promotion related signatures and immune-activation related signatures among three gene clusters. The upper and lower ends of the boxes represented interquartile range of values. The lines in the boxes represented median value, and small dots showed outliers. The asterisks represented the statistical p value. (*p < 0.05; **p < 0.01; ***p < 0.001). **Figure S6.** (A) Estimate score between High- and Low-risk groups. (B) Scale-free fit index for various soft-thresholding powers(β); Mean connectivity for various soft-thresholding powers. (C) function enrichment analysis. **Figure S7.** (A) Difference of Estimate score, immune score, stromal score between FRI-high and FRI-low group. (B) Gene expression of HLA gene sets between two distinct clusters. **Figure S8.** Association between 5-lncRNA riskscore subtypes and ICPs and ICD modulators. (A-E, G-J) Differential expression of ICP genes among the BCa riskscore subtypes in (A-E) TCGA and (G-J) IMvigor210 cohort. (F, K) Differential expression of ICD modulator genes among the BCa riskscore subtypes in (F) TCGA and (K) IMvigor210 cohorts. * p < 0.01, ** p < 0.001, *** p < 0.0001, and ****p < 0.00001. **Figure S9.** (A, B) Characteristics of tumor somatic mutation from 5-lncRNA signature in TCGA cohort.The mutation co-occurrence and exclusion analyses for high-(A) and low-risk(B) groups. Co-occurrence, green; Exclusion, yellow. **Figure S10.** (A) Correlation between TMB and riskscore. (B) Differences in riskscore among distinct TCGA phenotypes in IMvigor210 cohort. The lines in the boxes represented median value (p = 0.039, Kruskal–Wallis test) (C) Differences in riskscore among distinct immune phenotypes in IMvigor210 cohort. The lines in the boxes represented median value (p = 0.04, Kruskal–Wallis test). **Figure S11.** (A) Distribution of m6Ascore in distinct survival status groups. (B) Difference in risk score between distinct survival status groups. (p < 0.0001, Wilcoxon test) (C) The prognostic value of risk score and correlation between the clinicopathological features and risk score.

## Data Availability

All data generated and described in this article are available from the corresponding web servers, and are freely available to any scientist wishing to use them for noncommercial purposes, without breaching participant confidentiality. Further information is available from the corresponding author on reasonable request.

## Abbreviations

FeME: Ferroptosis induced tumor microenvironment
BCa: Bladder cancer
TME: Tumor microenvironment
lncRNAs: Long noncoding RNAs
GEO: Gene expression omnibus
OS: Overall survival
DFS: Disease-free survival
TCGA: The Cancer Genome Atlas
FRGs: Ferroptosis related genes
Fr-lncRNAs: Ferroptosis related lncRNAs
FRGs score: Ferroptosis related genes score
FRI: Ferroptosis risk index
CNV: Copy number variation
TIICs: Tumor immune infiltrating cells
DCs: Dendritic cells
MDSCs: Myeloid-derived suppressor cells
DEGs: Differentially expressed genes
EMT: Epithelial-mesenchymal transition
GS: Genome stable
GSVA: Gene set variation analysis
HR: Hazard ratios
ICB: Immunological checkpoint blockade
m6A: N6-methyladenosine
MSI: Microsatellite instability
PCA: Principal component analysis
ssGSEA: Single-sample gene-set enrichment analysis
KEGG: Kyoto Encyclopedia of Genes and Genomes
GO: Gene ontology
DMS: DNA methylation score
TIME: Tumor immune microenvironment
ICPs: Immune checkpoints
ICIs: Immune checkpoints inhibitors
ICD: Immunogenic cell death
TMB: Tumor mutation burden
