# Supplementary material for: Identification of mRNA vaccines and conserved ferroptosis related immune landscape for individual precision treatment in bladder cancer
Source: J Big Data. 2022 Jul 7;9(1):88. doi: 10.1186/s40537-022-00641-z (PMC9261131; doi:10.1186/s40537-022-00641-z)

**Additional Tables:**

**Table S1.** Univariate association of the 55-FrlncRNAs with overall survival in the meta-cohort

| id | HR | HR.95L | HR.95H | p-value |
| --- | --- | --- | --- | --- |
| PRDX6 | 1.541630944 | 1.359966096 | 1.74756266 | 1.79E-10 |
| EMP1 | 1.401686067 | 1.269383986 | 1.547777388 | 6.61E-10 |
| PRDX1 | 1.349132659 | 1.23511062 | 1.473680902 | 1.04E-09 |
| MAPK3 | 1.561793483 | 1.361182908 | 1.791969962 | 2.54E-09 |
| G6PD | 1.315681646 | 1.17316197 | 1.475515095 | 1.93E-07 |
| TFRC | 1.298071514 | 1.18064154 | 1.427181409 | 6.00E-07 |
| LAMP2 | 1.386782886 | 1.204896064 | 1.596126697 | 7.27E-07 |
| GCLM | 1.240820637 | 1.106777517 | 1.391097875 | 9.03E-07 |
| CRYAB | 1.118355743 | 1.030313235 | 1.213921675 | 9.30E-07 |
| SLC7A11 | 0.875550058 | 0.773371305 | 0.991228793 | 1.54E-06 |
| PGD | 1.32969 | 1.188096205 | 1.488158525 | 2.42E-06 |
| VDAC2 | 1.216899925 | 1.10062103 | 1.345463504 | 2.89E-06 |
| LOX | 1.180792426 | 1.080012719 | 1.290976234 | 8.53E-06 |
| SCD | 1.213642981 | 1.110906344 | 1.325880705 | 1.57E-05 |
| WIPI1 | 1.412352769 | 1.196916279 | 1.666566308 | 1.58E-05 |
| SLC3A2 | 1.536612018 | 1.300260329 | 1.815925965 | 1.65E-05 |
| ALOX5 | 0.83917557 | 0.767487599 | 0.917559631 | 1.69E-05 |
| SCP2 | 0.610367964 | 0.489746318 | 0.760698013 | 2.22E-05 |
| SLC39A14 | 1.264942572 | 1.115299575 | 1.434663605 | 5.29E-05 |
| JUN | 1.040777732 | 0.946591584 | 1.14433543 | 7.15E-05 |
| CAV1 | 1.101275733 | 1.011476282 | 1.199047631 | 7.64E-05 |
| NOX1 | 0.712571829 | 0.584651949 | 0.86848015 | 8.92E-05 |
| EIF4A1 | 1.159924473 | 1.061854625 | 1.267051771 | 9.55E-05 |
| GCH1 | 0.72980624 | 0.601167743 | 0.885970935 | 0.000206582 |
| SAT2 | 1.161062972 | 0.982149026 | 1.372568917 | 0.000217408 |
| FADS2 | 1.12931372 | 1.022487796 | 1.247300441 | 0.00032226 |
| VHL | 1.180570941 | 1.012933075 | 1.375952451 | 0.000376597 |
| TPD52 | 0.690637498 | 0.581743708 | 0.81991459 | 0.000719532 |
| SQLE | 1.096402688 | 0.97810664 | 1.229005922 | 0.000726691 |
| TMBIM4 | 1.197647475 | 1.056660937 | 1.35744535 | 0.000767007 |
| ITGB8 | 0.929713334 | 0.813518863 | 1.0625038 | 0.001206331 |
| SRC | 0.911449562 | 0.804832788 | 1.032189935 | 0.001225061 |
| FXN | 0.805270606 | 0.681029806 | 0.952176752 | 0.001635403 |
| FBXW7 | 0.747419903 | 0.588847467 | 0.94869477 | 0.001993414 |
| ACSL5 | 0.87714142 | 0.797819177 | 0.964350184 | 0.002261848 |
| ISCU | 1.091388326 | 0.906644865 | 1.313776236 | 0.003714709 |
| IDH1 | 0.889432854 | 0.80444488 | 0.983399634 | 0.003726435 |
| MAP3K5 | 0.92969564 | 0.827178591 | 1.044918221 | 0.004082136 |
| RNF113A | 0.976020994 | 0.809163389 | 1.177286311 | 0.006308759 |
| ALDH3A1 | 1.043293345 | 0.980506899 | 1.1101003 | 0.007206343 |
| IFNG | 0.848198203 | 0.735784142 | 0.97778703 | 0.007686893 |
| ATG4D | 0.782475358 | 0.630480717 | 0.971112469 | 0.009994667 |
| MYC | 1.009351318 | 0.924317451 | 1.102207994 | 0.010402607 |
| CDO1 | 1.037910209 | 0.897978725 | 1.199647132 | 0.012838666 |
| NEDD4 | 0.847880477 | 0.697947978 | 1.030021328 | 0.014146973 |
| ZEB1 | 0.935273334 | 0.799751454 | 1.093760074 | 0.01688885 |
| ACSL4 | 0.868083381 | 0.734179546 | 1.026409358 | 0.022533457 |
| SLC38A1 | 1.054881071 | 0.933113066 | 1.192539377 | 0.02762767 |
| DECR1 | 1.068766232 | 0.95056278 | 1.201668403 | 0.029613046 |
| AIFM2 | 0.886900398 | 0.731599053 | 1.075168583 | 0.031564855 |
| TGFBR1 | 0.873872786 | 0.725994661 | 1.051872261 | 0.031647133 |
| ABCC1 | 0.930429898 | 0.782945944 | 1.105695485 | 0.064368488 |
| EGFR | 1.060237953 | 0.944370182 | 1.190321909 | 0.065976148 |
| LATS2 | 1.058244943 | 0.87745659 | 1.27628235 | 0.076282878 |
| SLC2A14 | 1.053196773 | 0.853048091 | 1.300305872 | 0.318272995 |

**Table S2.** Multivariate cox regression analysis of the 5-FrlncRNAs with overall survival in the TCGA train cohort

| id | coef | HR | HR.95L | HR.95H | pvalue |
| --- | --- | --- | --- | --- | --- |
| LINC01426 | 0.265400655 | 1.303953306 | 1.058429956 | 1.606430557 | 0.012649627 |
| LINC01098 | 1.153344469 | 3.168773071 | 1.748660807 | 5.742178664 | 0.000143293 |
| C6orf99 | 0.36167568 | 1.435733229 | 1.100267952 | 1.873479911 | 0.007728447 |
| LINC01614 | 0.035157161 | 1.035782481 | 1.009199951 | 1.0630652 | 0.00804121 |
| ST7-OT4 | 5.16519574 | 175.0717238 | 20.9043345 | 1466.208287 | 1.90E-06 |

**Table S3.** Univariate association of the 5-lncRNAs ferroptosis-related signature with overall survival in the three sets

| Parameters | Training set | | Internal test set | | IMvigor210 cohort | |
| --- | --- | --- | --- | --- | --- | --- |
|  | HR (95%CI) | p | HR (95%CI) | p | HR (95%CI) | p |
| Age (≥60 years *vs*. <60 years) | 1.032 (1.016-1.048) | <0.001 | 1.118 (1.025–1.242) | 0.012 | 1.162 (0.983–1.350) | 0.004 |
| Gender (male *vs*. female) | 0.908 (0.650-1.268) | 0.572 | 1.238(0.721–1.476) | 0.855 | 1.375(1.181–1.842) | 0.962 |
| T | 1.219 (1.010-1.471) | 0.039 | 1.991(1.704–2.613) | <0.001 | NA | NA |
| N | 1.229 (1.110-1.360) | <0.001 | 1.522(1.401–3.514) | <0.001 | NA | NA |
| M | 1.199 (1.030-1.396) | 0.019 | 2.113(1.967–4.532) | <0.001 | NA | NA |
| stage | 1.735(1.425-2.112) | <0.001 | 1.689(1.247-2.046) | <0.001 | 2.001(1.624-2.752) | <0.001 |
| 5-lncRNAs signature (high *vs*. low risk) | 1.564 (1.251–1.956) | <0.001 | 1.247(1.105–1.417) | <0.001 | 1.623 (1.203–1.996) | <0.001 |

**Table S4.** Multivariate Cox regression analysis of the 5-lncRNAs-ferroptosis-related signature with overall survival in the three sets

| Parameter | Training set | | Internal test set | | IMvigor210 cohort | |
| --- | --- | --- | --- | --- | --- | --- |
|  | HR (95%CI) | p | HR (95%CI) | p | HR (95%CI) | p |
| Age (≥60 years vs. <60 years) | 1.033 (1.016–1.049) | 0.001 | 1.109 (1.010–1.152) | 0.003 | 1.263 (1.033–1.388) | 0.080 |
| Sex (male *vs*. female) | 0.900 (0.644–1.259) | 0.539 | 1.003 (0.688–1.598) | 0.477 | 0.893 (0.594–1.127) | 0.35 |
| T | 1.214 (1.108–1.282) | 0.075 | 1.284 (0.983–1.736) | 0.121 | NA | NA |
| N | 1.196 (1.058–1.353) | 0.004 | 2.203 (1.304–4.332) | 0.028 | NA | NA |
| M | 1.152 (0.984–1.348) | 0.079 | 1.244 (1.144–4.822) | <0.001 | NA | NA |
| stage | 1.474(1.182-1.837) | <0.001 | 1.301(1.026-2.388) | <0.001 | 2.211(2.196-3.366) | <0.001 |
| 5-lncRNAs signature (high *vs*. low risk) | 1.513 (1.195–1.917) | <0.001 | 1.096 (1.032–1.164) | 0.002 | 1.374 (1.112–2.210) | <0.001 |

**Table S5.** Univariate and Multivariate Cox regression analysis of the conserved 5-lncRNAs-ferroptosis-related signature with overall survival in TCGA cohort

| Parameters | **Univariate** | | **Multivariate** | |
| --- | --- | --- | --- | --- |
|  | HR (95%CI) | p | HR (95%CI) | p |
| Age (≥60 years *vs*. <60 years) | 1.026 (1.008-1.044) | 0.004 | 1.304 (1.116–1.623) | <0.001 |
| Gender (male *vs*. female) | 1.294 (0.866-1.802) | 0.235 | 1.289(1.143–1.820) | 0.275 |
| Grade | 1.361 (0.969-1.911) | 0.075 | 1.463(1.175–1.831) | 0.066 |
| Stage | 1.534 (1.241-1.896) | <0.001 | 1.946(1.364–1.824) | 0.024 |
| TMB | 0.628(0.514-0.768) | <0.001 | 0.858(0.441–0.996) | 0.014 |
| m6Ascore | 1.516(1.227-1.874) | <0.001 | 1.256(1.120-1.675) | 0.042 |
| DMSscore | 1.385(1.109-1.728) | 0.004 | 1.455(1.206-1.811) | 0.019 |
| 5-lncRNAs signature (high *vs*. low risk) | 1.048 (1.033–1.062) | <0.001 | 1.378(1.125–1.501) | <0.001 |

**Additional Figures**：

Figure S1. Identification of tumor antigens associated with BCa prognosis. (A) Kaplan-Meier curves showing OS and DFS of BCa patients stratified on the basis of FADS2, SLC3A2, SCD, TFRC, SQLE,and G6PD expression levels. (B) Difference of mRNA expression level between four CNV types in TCGA cohort. (C) Kaplan-Meier curves showing OS of BCa patients stratified on the basis of FADS2, SLC3A2, SCD, TFRC, SQLE,and G6PD expression levels in meta cohort.


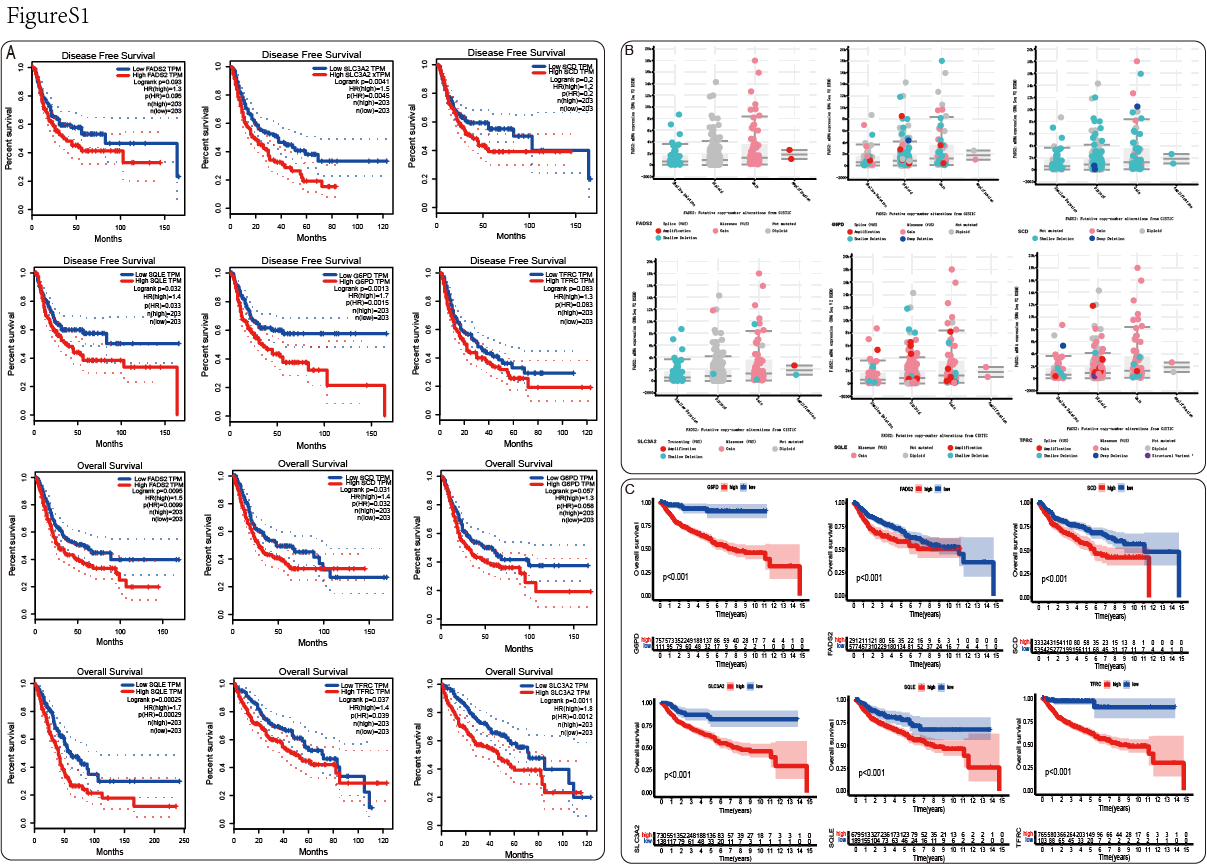


Figure S2. (A) The interaction between ferroptosis regulators in bladder cancer. The circle size represented the effect of each regulator on the prognosis, and the range of values calculated by Log-rank test was p < 0.001, p < 0.01, p < 0.05 and P < 0.1, respectively. (B) The mutation co-occurrence and exclusion analyses between FBXW7 and other ferroptosis regulators. Co-occurrence, green; Exclusion, yellow. (C) The heatmap of unsupervised clustering of 55 ferroptosis regulators in the combined bladder cancer cohorts. The FRGs cluster, tumor stage, survival status and age were used as patient annotations. Red represented high expression of regulators and blue represented low expression.


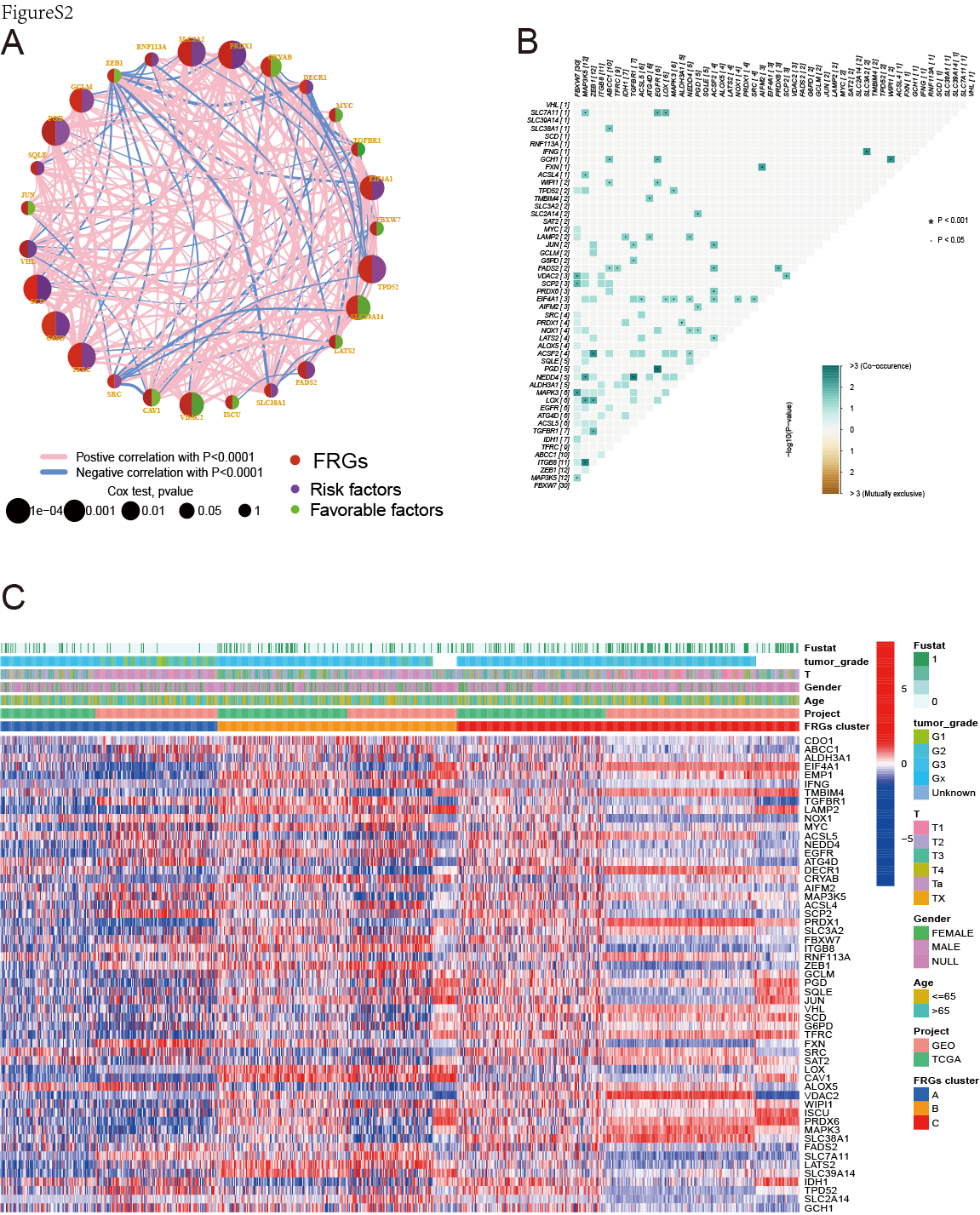


Figure S3. (A-D) Consensus matrices of the IMvigor210 cohort for k = 2 - 5. (E) Unsupervised clustering of 55 ferroptosis regulators in the IMvigor210 bladder cancer cohort. The FRGs cluster, immune phenotype, overall response, tumor stage, survival status and age were used as patient annotations. Red represented high expression of regulators and blue represented low expression. (F) Principal component analysis for the transcriptome profiles of three ferroptosis regulation patterns, showing a remarkable difference on transcriptome between different regulation patterns


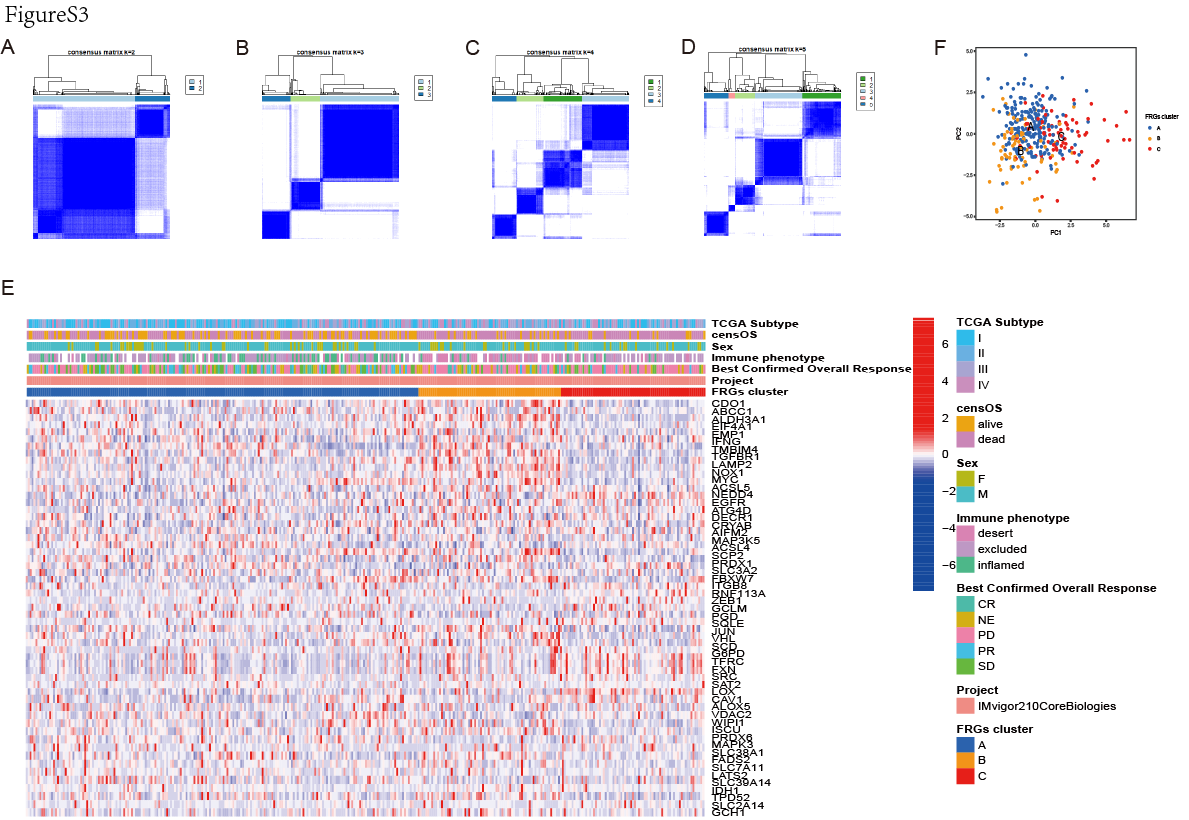


Figure S4. Construction of ferroptosis related genes (FRGs) score. (A) Principal component analysis between different regulation patterns. (B) 3310 ferroptosis phenotype-related genes shown in venn diagram. (C-D) Functional annotation using GO and KEGG enrichment analysis. (E) Consensus matrices of the combined cohort for k = 3. (F) Survival analyses for the three ferroptosis phenotype-related gene cluster. (G) The expression of 55 ferroptosis regulators in three gene cluster. (H) Survival analyses of FRGs score (I) ggalluvial diagram plot. (J) Correlations between FRGs score and the known gene signatures. (K) Differences of FRGs score among different clusters. (P < 0.001).


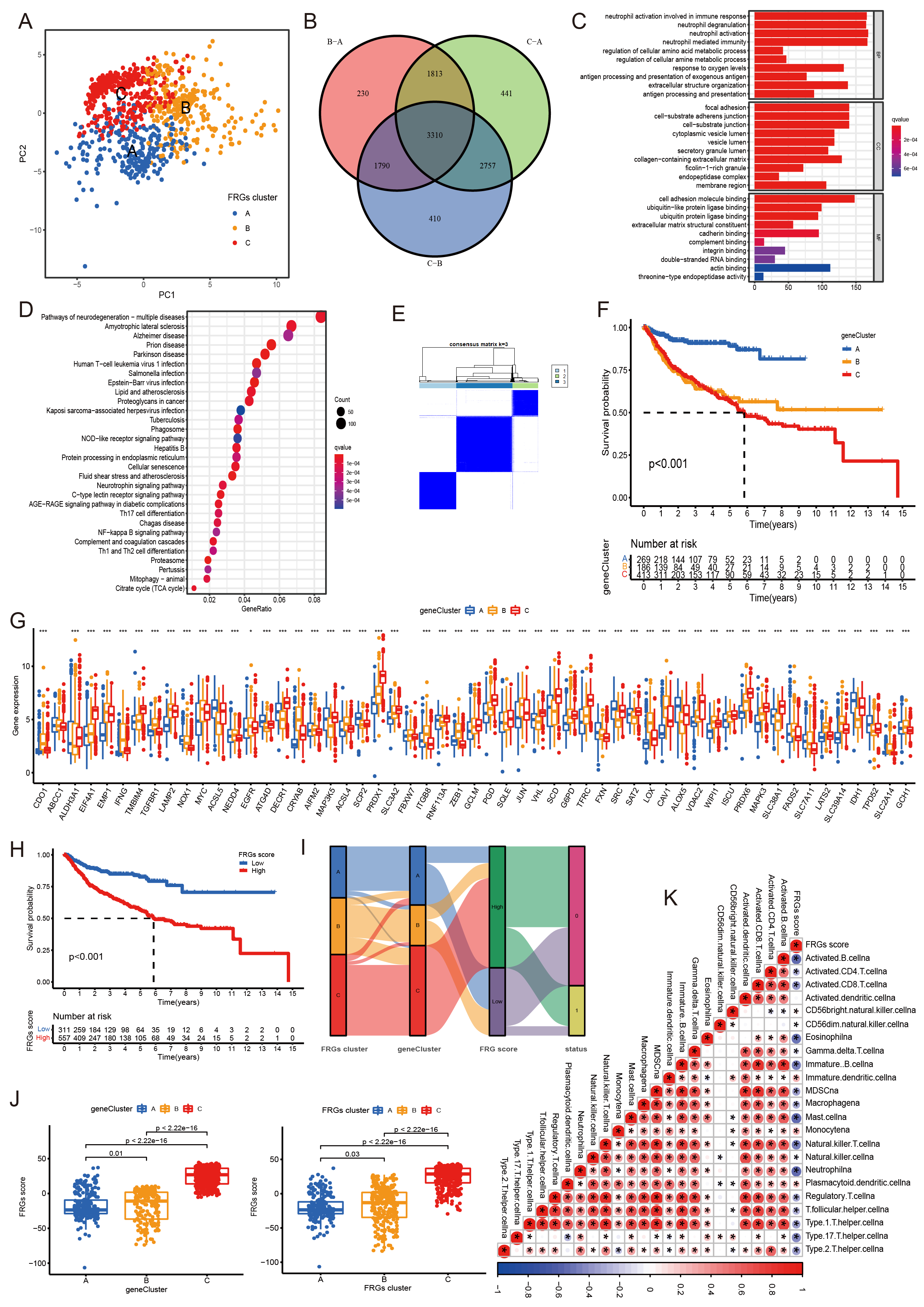


Figure S5. Characteristics of cytokine transcriptome, chemokine transcriptome and known signatures in distinct gene clusters (A) Difference in the immune-activation related gene expression among three gene clusters. (B) Difference in the immune-checkpoint related gene expression among three gene clusters. (C) Difference in the TGFβ- EMT pathway-related gene expression among three gene clusters. (D) Difference in the expression of known signatures including stromal-activation related signatures, tumor-promotion related signatures and immune-activation related signatures among three gene clusters. The upper and lower ends of the boxes represented interquartile range of values. The lines in the boxes represented median value, and small dots showed outliers. The asterisks represented the statistical p value. (*P < 0.05; **P < 0.01; ***P < 0.001).


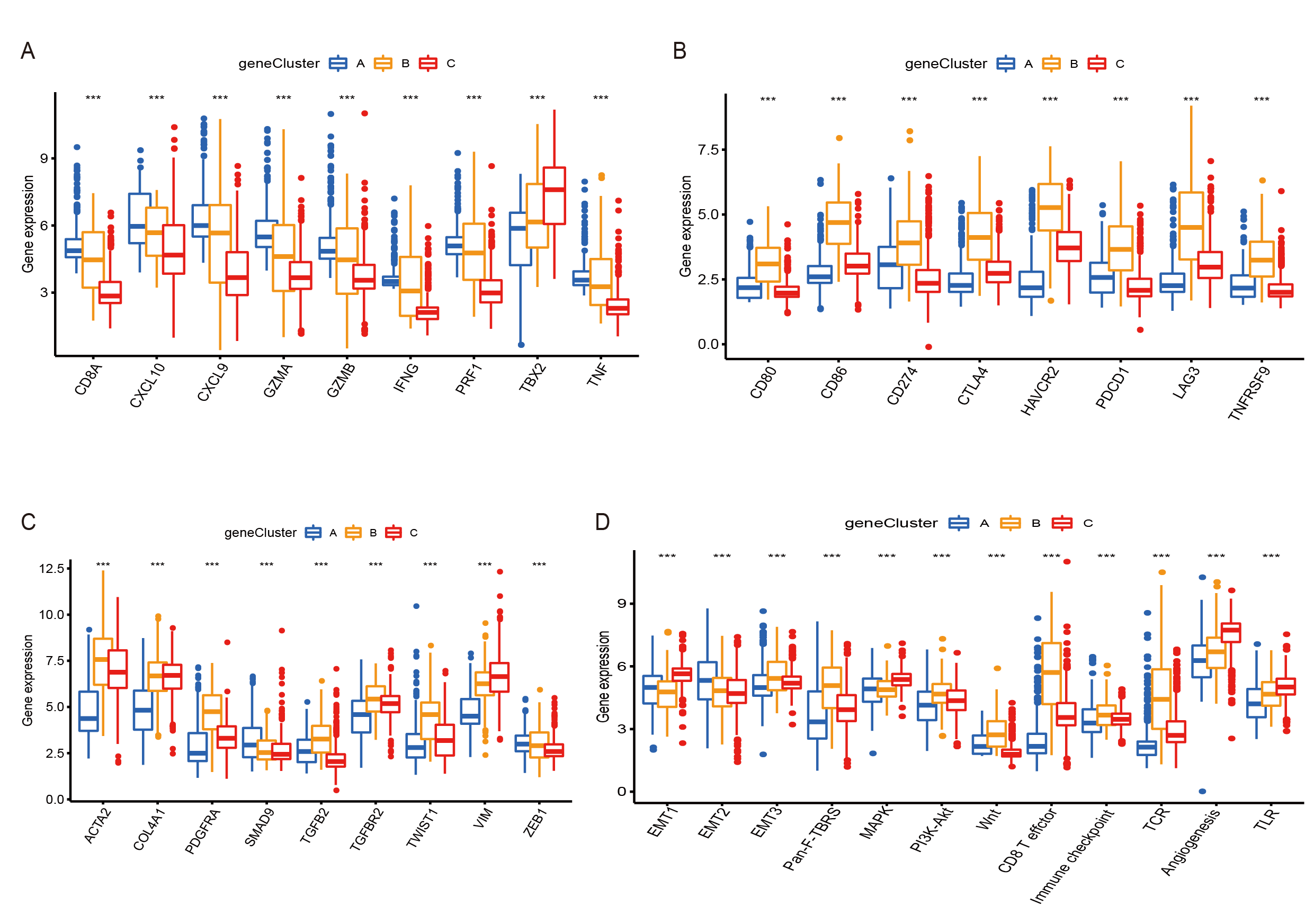


Figure S6. (A) Estimate score between High- and Low-risk groups. (B) Scale-free fit index for various soft-thresholding powers(β); Mean connectivity for various soft-thresholding powers. (C) function enrichment analysis.


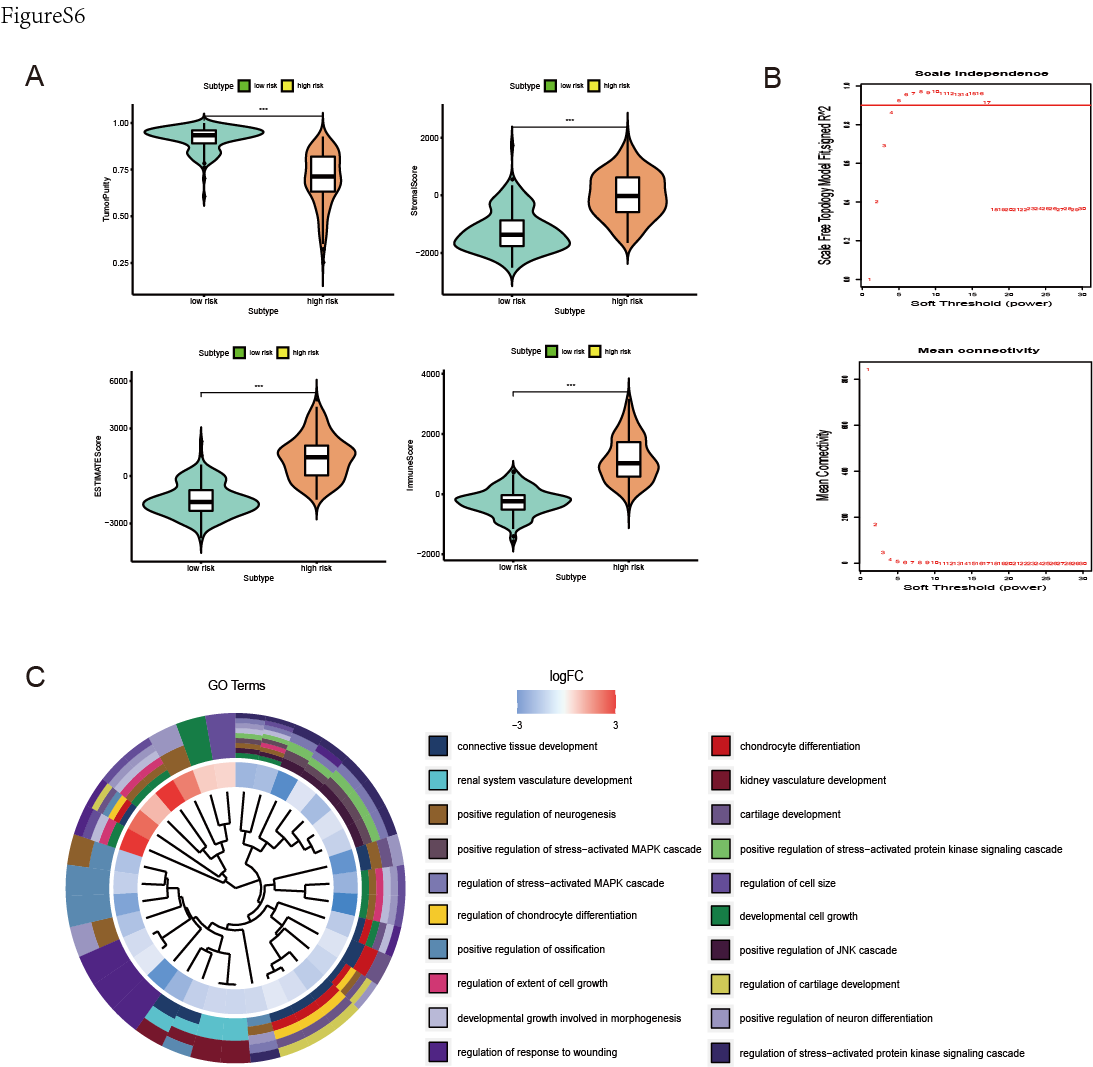


Figure S7. (A) Difference of Estimate score, immune score, stromal score between FRI-high and FRI-low group. (B) Gene expression of HLA gene sets between two distinct clusters.


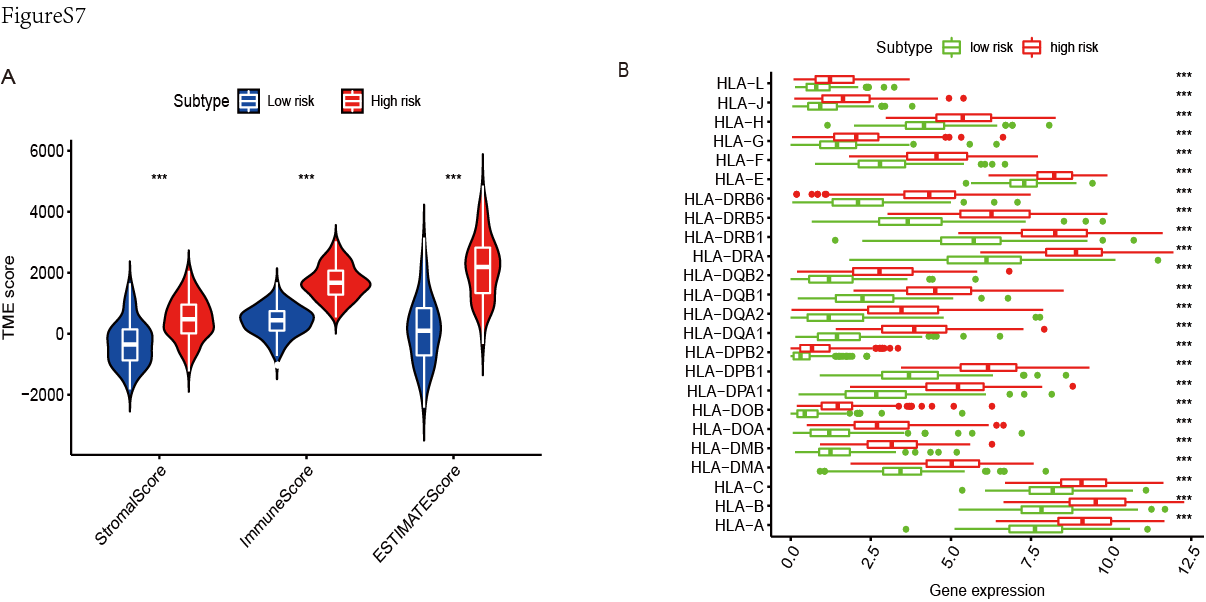


Figure S8. Association between 5-lncRNA riskscore subtypes and ICPs and ICD modulators. (A-E, G-J) Differential expression of ICP genes among the BCa riskscore subtypes in (A-E) TCGA and (G-J) IMvigor210 cohort. (F, K) Differential expression of ICD modulator genes among the BCa riskscore subtypes in (F) TCGA and (K) IMvigor210 cohorts. * p < 0.01, ** p < 0.001, *** p < 0.0001, and ****p < 0.00001.


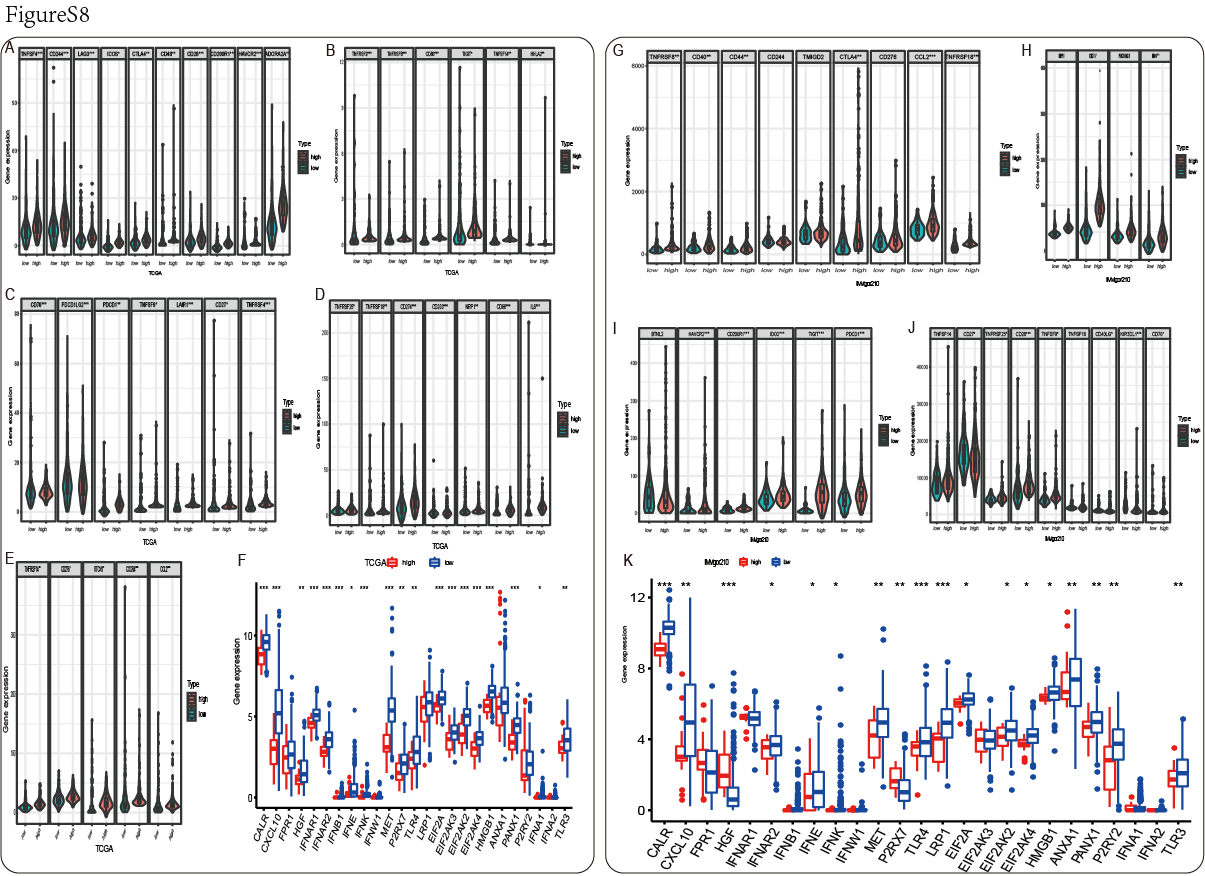


Figure S9. (A, B) Characteristics of tumor somatic mutation from 5-lncRNA signature in TCGA cohort.The mutation co-occurrence and exclusion analyses for high-(A) and low-risk(B) groups. Co-occurrence, green; Exclusion, yellow.


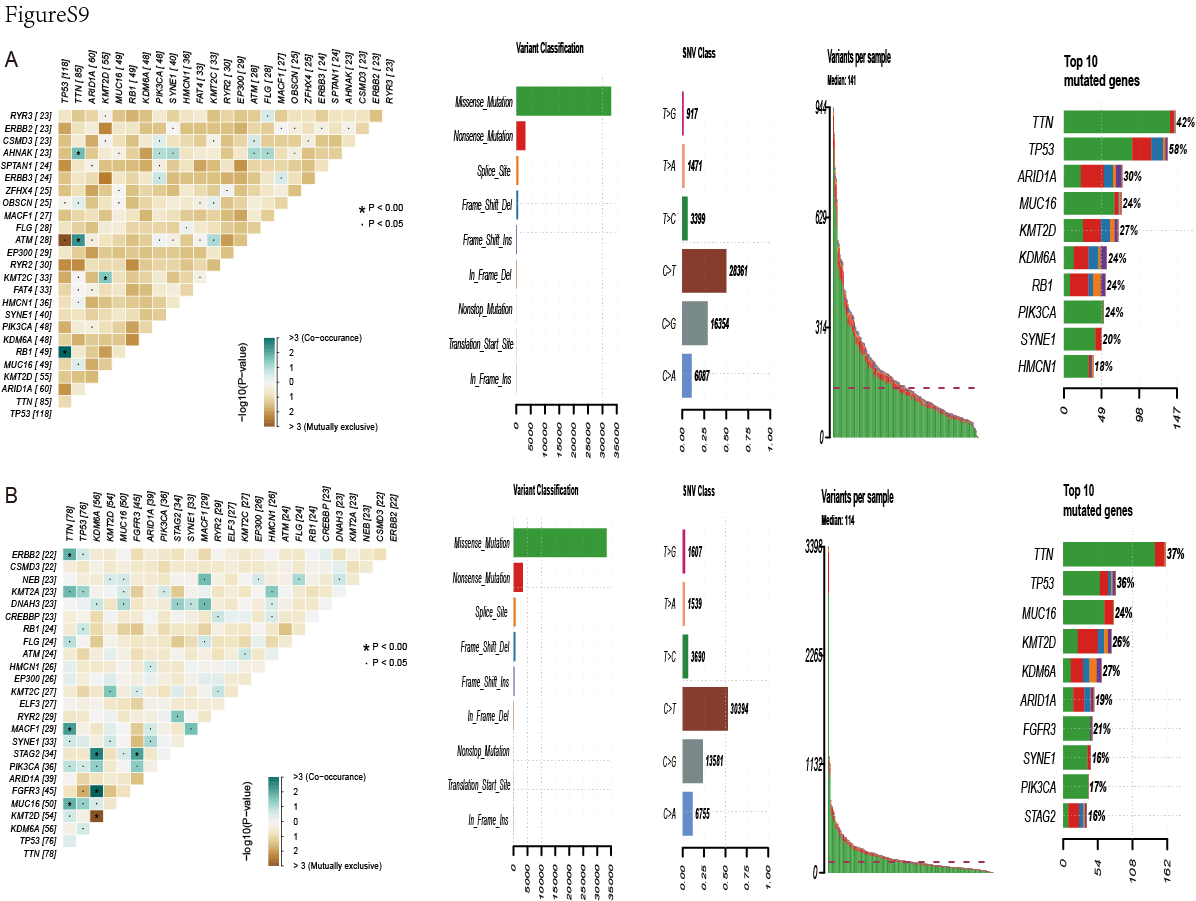


Figure S10. (A) Correlation between TMB and riskscore. (B) Differences in riskscore among distinct TCGA phenotypes in IMvigor210 cohort. The lines in the boxes represented median value (p = 0.039, Kruskal-Wallis test) (C) Differences in riskscore among distinct immune phenotypes in IMvigor210 cohort. The lines in the boxes represented median value (p = 0.04, Kruskal-Wallis test).


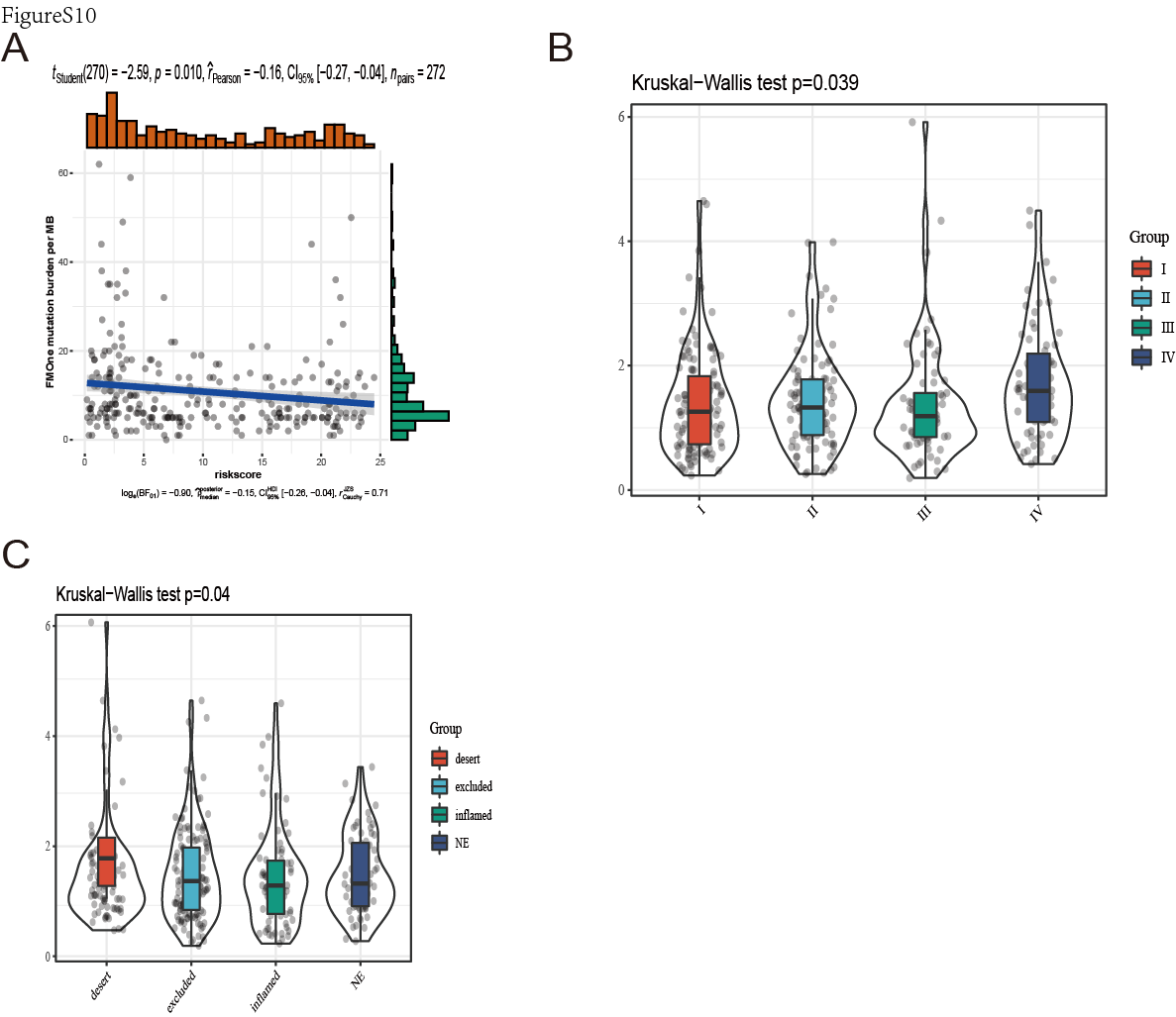


Figure S11. (A) Distribution of m6Ascore in distinct survival status groups. (B) Difference in risk score between distinct survival status groups. (p<0.0001, Wilcoxon test) (C) The prognostic value of risk score and correlation between the clinicopathological features and risk score.


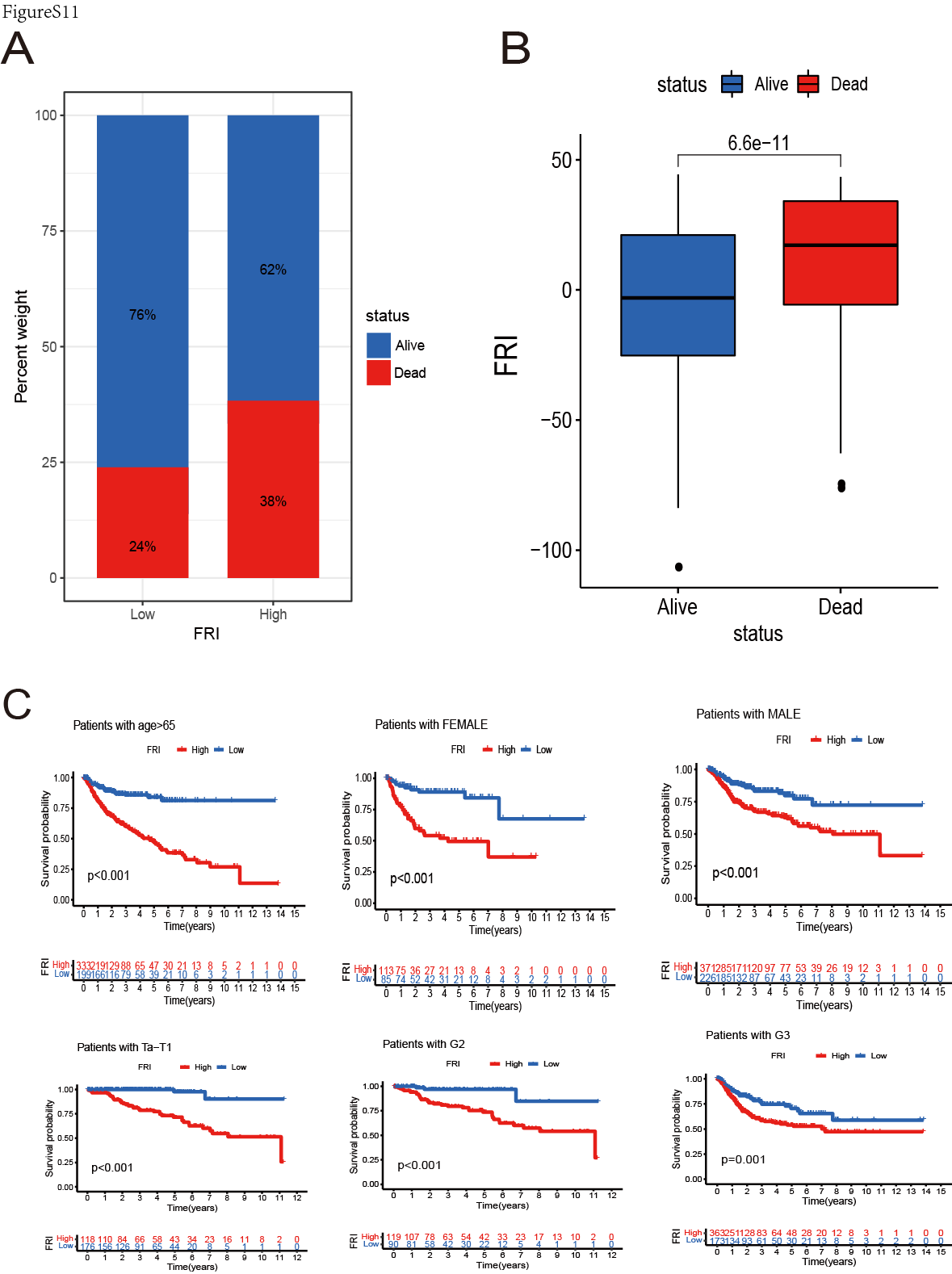

Supplement: Supplementary file 1 — Additional file 1: Table S1. Univariate association of the 55-FrlncRNAs with overall survival in the meta-cohort. Table S2. Multivariate cox regression analysis of the 5-FrlncRNAs with overall survival in the TCGA train cohort. Table S3. Univariate association of the 5-lncRNAs ferroptosis-related signature with overall survival in the three sets. Table S4. Multivariate Cox regression analysis of the 5-lncRNAs-ferroptosis-related signature with overall survival in the three sets. Table S5. Univariate and Multivariate Cox regression analysis of the conserved 5-lncRNAs-ferroptosis-related signature with overall survival in TCGA cohort. Figure S1. Identification of tumor antigens associated with BCa prognosis. (A) Kaplan–Meier curves showing OS and DFS of BCa patients stratified on the basis of FADS2, SLC3A2, SCD, TFRC, SQLE,and G6PD expression levels. (B) Difference of mRNA expression level between four CNV types in TCGA cohort. (C) Kaplan–Meier curves showing OS of BCa patients stratified on the basis of FADS2, SLC3A2, SCD, TFRC, SQLE,and G6PD expression levels in meta cohort. Figure S2. (A) The interaction between ferroptosis regulators in bladder cancer. The circle size represented the effect of each regulator on the prognosis, and the range of values calculated by Log-rank test was p < 0.001, p < 0.01, p < 0.05 and p < 0.1, respectively. (B) The mutation co-occurrence and exclusion analyses between FBXW7 and other ferroptosis regulators. Co-occurrence, green; Exclusion, yellow. (C) The heatmap of unsupervised clustering of 55 ferroptosis regulators in the combined bladder cancer cohorts. The FRGs cluster, tumor stage, survival status and age were used as patient annotations. Red represented high expression of regulators and blue represented low expression. Figure S3. (A-D) Consensus matrices of the IMvigor210 cohort for k = 2—5. (E) Unsupervised clustering of 55 ferroptosis regulators in the IMvigor210 bladder cancer cohort. The FRGs cluster, immune phenotype, over [file 40537_2022_641_MOESM1_ESM.doc]
